# Effect of Arylazo Sulfones on DNA: Binding, Cleavage, Photocleavage, Molecular Docking Studies and Interaction with A375 Melanoma and Non-Cancer Cells

**DOI:** 10.3390/ijms24031834

**Published:** 2023-01-17

**Authors:** Chrysoula Mikra, Achilleas Mitrakas, Virginia Ghizzani, Katerina R. Katsani, Maria Koffa, Michael Koukourakis, George Psomas, Stefano Protti, Maurizio Fagnoni, Konstantina C. Fylaktakidou

**Affiliations:** 1 Laboratory of Organic Chemistry, Faculty of Chemistry, Aristotle University of Thessaloniki, 54124 Thessaloniki, Greece; 2 Laboratory of Cellular Biology and Cell Cycle, Molecular Biology and Genetics Department, Democritus University of Thrace, University Campus, Dragana, 68100 Alexandroupolis, Greece; 3 Department of Radiotherapy and Oncology, Democritus University of Thrace, University General Hospital of Alexandroupolis, 68100 Alexandroupolis, Greece; 4 PhotoGreen Lab, Department of Chemistry, University of Pavia, V. Le Taramelli 12, 27100 Pavia, Italy; 5 Laboratory of Biochemistry and Molecular Virology, Molecular Biology and Genetics Department, Democritus University of Thrace, Dragana, 68100 Alexandroupolis, Greece; 6 Laboratory of Inorganic Chemistry, Faculty of Chemistry, Aristotle University of Thessaloniki, 54124 Thessaloniki, Greece

**Keywords:** arylazo sulfones, DNA binding, DNA cleavage, DNA photocleavage, A375 melanoma cells, molecular docking, radicals, N–S bond homolysis

## Abstract

A set of arylazo sulfones, known to undergo N–S bond cleavage upon light exposure, has been synthesized, and their activity in the dark and upon irradiation towards DNA has been investigated. Their interaction with calf-thymus DNA has been examined, and the significant affinity observed (most probably due to DNA intercalation) was analyzed by means of molecular docking “in silico” calculations that pointed out polar contacts, mainly via the sulfonyl moiety. Incubation with plasmid pBluescript KS II revealed DNA cleavage that has been studied over time and concentration. UV-A irradiation considerably improved DNA damage for most of the compounds, whereas under visible light the effect was slightly lower. Moving to in vitro experiments, irradiation was found to slightly enhance the death of the cells in the majority of the compounds. Naphthylazosulfone **1** showed photo-disruptive effect under UV-A irradiation (IC_50_ ~13 μΜ) followed by derivatives **14** and **17** (IC_50_ ~100 μΜ). Those compounds were irradiated in the presence of two non-cancer cell lines and were found equally toxic only upon irradiation and not in the dark. The temporal and spatial control of light, therefore, might provide a chance for these novel scaffolds to be useful for the development of phototoxic pharmaceuticals.

## 1. Introduction

A wide variety of small organic molecules have been designed and investigated for their interaction with structural features of DNA, aiming to target the transcriptional machinery of cancer cells and lead to apoptosis. Indeed, DNA modification may inhibit cancer progression, and this can be achieved via several pathways, including hydrolysis of phosphodiesters and oxidation of the deoxyribose sugar or the nucleotide bases [1,2]. In this context, the affinity of a small molecule with DNA is examined as the initial step [3] by means of spectroscopic analyses able to provide an evaluation of the changes in the DNA moiety while interacting with the examined compound [4]. Thus, intercalation of the “host” with DNA, minor and major groove binding can be revealed, indicating its important physicochemical approach that is a prerequisite for possible effective damage.

Although most DNA-cleaving compounds are metal complexes, a significant number of research publications are devoted to organic molecules, also referred to as artificial “metal-free nucleases”, aiming to identify differentiated mechanisms of action. Such compounds exhibit very diversified molecular structures, including, among the recent ones, simple oximes and hydroxylamines [5], coumarin oxime ethers [6], imidazo-phenanthrolines [7] and their carbohydrate conjugates [8], indolo-pyrimidines [9], pyridine [10] and naphthoquinone thiazole hybrids [11] and benzothiazole derivatives [12,13], bis- and tetrakis-1,2,3-triazole derivatives [14], naphthalenophanes [15], selenylated oxadiazoles [16], 2-styryl-4-aminoquinazoline [17], calixarene-benzazole [18] and indolyl derivatives [19], azaenedienes [20], and the natural product Shishjimicin A [21]. 

The challenge to find DNA binding molecules that do not interfere with the functions of normal cells and/or to overcome multidrug resistance to chemotherapeutics is the most desirable goal. In this direction, due to the high spatial and temporal control, photochemotherapy [22,23,24] and photodynamic therapy [25,26,27,28] allow the minimally invasive treatment of several kinds of cancer and other nonmalignant diseases. Such impressive selectivity is due to the role of light, which acts as a counterpart to small organic molecules that can be excited by the energy offered by light and affect the biological target. Thus, a photosensitizer activatable under UV-B and UV-A irradiation is the requirement in photochemotherapy and a photosensitizer in combination with UV-A/visible and NIR irradiations with concomitant participation of oxygen reactive species (mainly singlet oxygen) characterize the photodynamic one. 

However, to overcome multidrug resistance to chemotherapeutics, combinations of photochemotherapy and chemotherapy were investigated for the treatment of cutaneous T-cell lymphoma [29], whereas combinations of chemotherapy and photodynamic therapy were applied to study the synergistic effects in various cancer cells [30] and for better therapeutic efficacy in prostate [31] and breast cancer [32], advanced gastric adenocarcinoma [33], etc. Additionally, photosensitizers are increasingly used for the inactivation of bacteria and other microorganisms [34,35,36], with the scientist suspecting effectiveness that will not reach the dead ends of antibiotics because of the multiple biological targets of the process [37,38]. 

Recently, the concept of a *dyedauxiliary* group was introduced [39]. This moiety may be incorporated within an organic molecule to induce a bathochromic shift and make it colored. At the same time, these groups bear a chemical bond that is labile upon visible (or UV) light excitation, thus causing the photorelease of reactive intermediates (e.g., radicals) [39,40].

**Figure 1:**
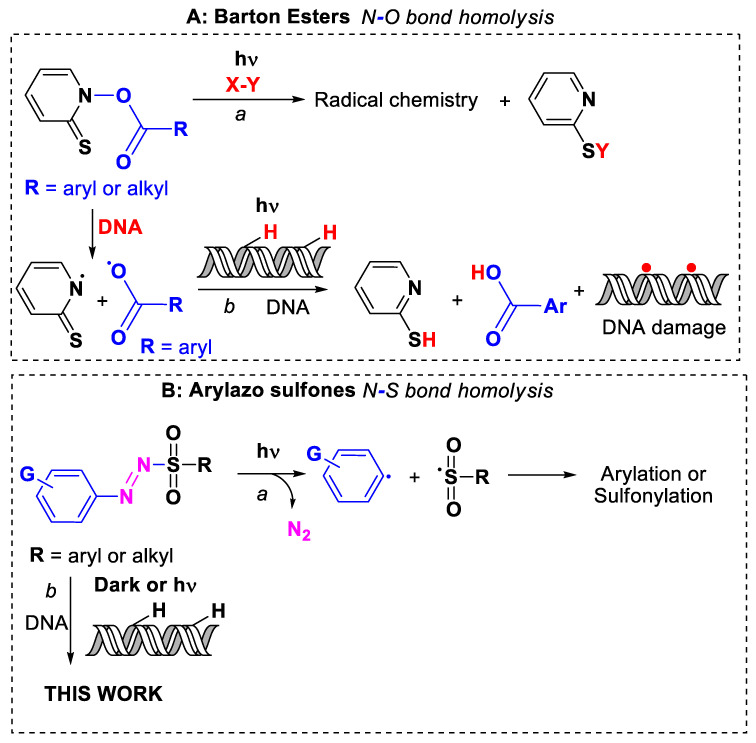
Light-induced homolysis: (**A**) of an N–O bond in Barton esters in synthesis (path *a*) and in chemical biology (path *b*); (**B**) of an N–S bond in arylazo sulfones in synthesis (path *a*) and in chemical biology (path *b*, this work).

Barton esters, which hold a photolabile N–O bond, have been employed in radical addition chain reactions for the synthesis of natural products as well as in the C–X (X = sulfur, selenium, halogen, nitrogen) bond formation [41,42,43,44] (Figure 1A), path *a*). The DNA photocleavage induced by these *O*-acyl thiohydroxamate esters has been previously investigated [45,46,47]. Indeed, the N–O bond homolysis of Barton esters generated aroyloxyl (oxygen-centered) radicals (Figure 1A, path *b*) known to attack a thymidine model, providing, along with the photobiological experiments, evidence of the efficiency of the oxygen-centered reactive intermediates under chemical conditions [48]. In analogy with *O*-acyl thiohydroxamate esters, *N*-aroyloxynaphthalimides exhibited good intercalation and DNA photocleavage upon UV-A exposure [49,50]. 

The concept of using photolabile N–O bonds is general, as in the case of functionalized oximes that are employed in chemistry for the creation of carbon, nitrogen, and oxygen-centered radicals [51,52,53,54] and as photoinitiators in polymerizations [55]. Oxime carboxylates have been applied to the DNA photocleavage of various substrates under UV-B and UV-A irradiation [56,57,58,59,60], along with oxime sulfonates [61,62] and oxime carbamates [63]. It should be noted that all the above-described compounds showed affinity to DNA, which is a prerequisite for DNA photocleavage, and they are classified as “true” DNA photo-cleavers because they show no evidence of DNA cleavage in the dark.

Recently, our team has extensively studied a class of colored shelf-stable derivatives containing a *dyedauxiliary* group, namely arylazo sulfones (Ar-N = N-SO_2_-R’). Such derivatives underwent the N–S bond homolysis upon visible light irradiation (Figure 1B, path *a*) and the ensuing loss of a molecule of nitrogen facilitates the formation of Ar˙ and R’-S˙O_2_ reactive radicals that have been exploited in the preparation of, among the others, arylstannanes [64], (hetero)arylphosphonates [65], aryl selenides and tellurides [66], symmetrical (hetero)biaryls [67], arylboronates [68], sulfonyl fluorides [69], stilbenes and vinyl sulfones [70], trifluoromethylthiolates [71], and (*E*)-vinyl sulfones [72]. Additionally, arylazo sulfones were able to initiate the polymerization of a broad range of electron-poor olefins [73], or to cause covalent functionalization (arylation) of reduced graphene oxide nanosheets [74] and simultaneous photografting of both aryl and methanesulfonyl groups on a gold surface [75]. 

**Figure 2:**
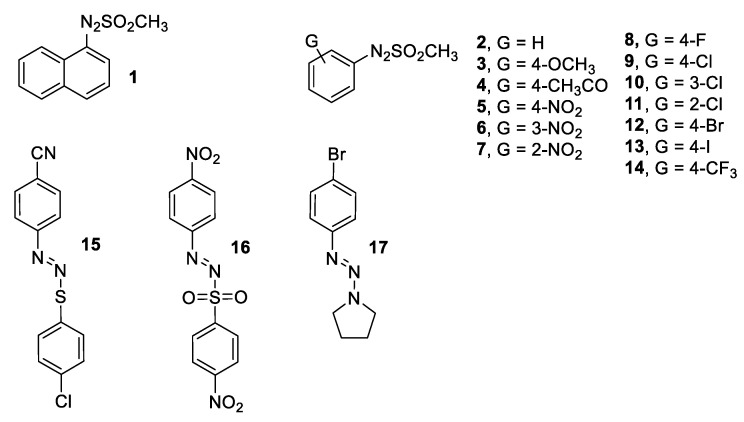
Arylazo sulfones (**1**–**14, 16**), arylazo sulfide (**15**), and triazene (**17**) prepared and investigated in this work.

We were eager, within this manuscript, to investigate whether the rich photochemistry of arylazo sulfones might have an effect on biomolecules and live cells, which are, to the best of our knowledge, yet totally unexplored. For this reason, using a well-established methodology, we have synthesized a set of arylazo sulfones (**1**–**14**, **16**, Figure 2) with compounds bearing various electron-donating and withdrawing groups on the aryl group. The results have been compared with those obtained with compounds bearing a different azo moiety, including an arylazo sulfide (**15**) and a triazene (**17**). A DNA binding profile of the total set of seventeen compounds has been studied using calf-thymus (CT) DNA and UV–vis and fluorescence spectroscopy as well as viscosity experiments. The DNA cleavage (in dark) and photocleavage (under UV-A and visible light irradiations) were studied using plasmid DNA pBluescript SK II and visualized via agarose gel electrophoresis (Figure 1B, path *b*). The highly malignant A375 melanoma cells were the model cells that provided initial results on cell cultures under dark, UV-A [76,77], and visible light exposure. Additionally, two non-cancer cell lines have been used as a control for the activity; HFL1, a fibroblast cell line that was isolated from the lung of a white, normal embryo, and HaCaT cell lines, human immortalized keratinocytes.

## 2. Results and Discussion

### 2.1. Synthesis and Characterization of Arylazo Sulfones

A representative set of arylazo sulfones (mesylates **1**–**14** and *p*-nitrophenylsulfonate **16**, Figure 2) have been prepared according to a reported procedure [64]. As hinted at above, two further derivatives where the sulfone moiety has been replaced by a thioaryl group and a piperidine moiety (azosulfide **15** and triazene **17**, respectively) were likewise prepared for the sake of comparison (copies of the NMR spectra of compounds **15** and **16** are available in Supporting Information, S.1, part S.1.a,b and S.1.c,d, respectively).

The seventeen compounds were preliminarily divided into four groups (A–D). Group A contained hydrogen, carbon, and oxygen substituents on the aromatic moiety of arylazo mesylates (**1**–**4**), Group B contained all nitro-substituted derivatives (**5**–**7**) and *p*-nitrophenylazo (*p*-nitrophenyl)sulfone (**16**), Group C contained all halogenated compounds (**8**–**13**), and Group D contained the remaining compounds (**14**, **15**, and **17**). 

### 2.2. CT DNA Binding Studies of Arylazo Sulfones

The interaction of compounds **1**–**17** with calf-thymus DNA (CT DNA) was investigated in vitro by UV–vis spectroscopy and viscosity measurements and via their ability to displace ethidium bromide (EB) from the EB-DNA adduct, which was examined by fluorescence emission spectroscopy.

**Figure 3:**
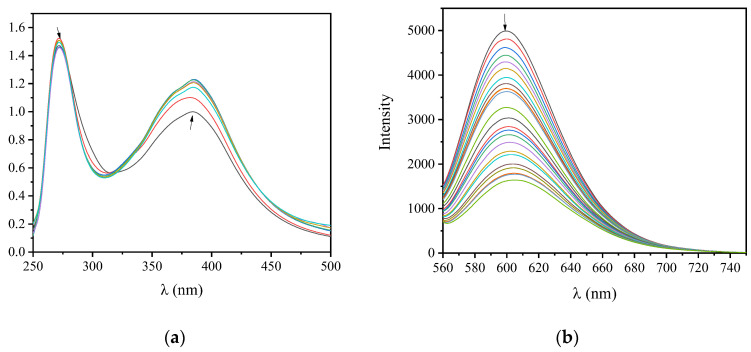
(**a**) UV–vis spectra of compound **1** (2.5 × 10^−4^ M) in DMSO in the presence of increasing amounts of CT DNA. The arrows show the changes upon increasing amounts of CT DNA. (**b**) Fluorescence emission spectra (λ_exc_ = 540 nm) for EB-DNA conjugate ([EB] = 20 μM, [DNA] = 26 μM) in buffer solution (150 mM NaCl and 15 mM trisodium citrate at pH = 7.0) in the presence of increasing amounts of compound **9**. The arrow shows the changes in intensity upon increasing amounts of compound 9.

The structural changes induced by the interaction of CT DNA with the examined compounds have been investigated by means of UV–vis spectroscopy, which was exploited to measure DNA-binding constants (K_b_). In most cases, the bands observed in the UV–vis spectra of the compounds (see the representative case of compound **1** shown in Figure 3a) exhibited, in the presence of increasing amounts of CT DNA, slight hyperchromism or even hypochromism accompanied by a slight red-shift (Table 1). These features may be attributed to the interaction of the compounds with CT DNA, whereas the interaction mode may not be safely interpreted, necessitating the performance of other experiments such as DNA-viscosity measurements.

The values of K_b_ of the compounds (Table 1) were determined with the Wolfe–Shimer equation (SI, Part S.2.1, Equation (1)) [78] and the plots [DNA]/(ε_A_–ε_f_) versus [DNA] (SI, Figure Part S.3). The K_b_ of the compounds **1**–**17** (in the order 10^5^–10^6^ M^−1^) are higher than that of the classical intercalator EB (=1.23(±0.07) × 10^5^ M^−1^) [79] and show the magnitude of their interaction with CT DNA.

Among the compounds of Group A, compound **4** (the *p*-CH_3_CO-substituted) presents the highest K_b_ value (=6.08(±0.15) × 10^5^ M^−1^) whereas among the NO_2_ derivatives in Group B, the *m*-NO_2_-isomer **6** exhibited the strongest affinity (K_b_ = 2.87(±0.10) × 10^7^ M^−1^) and the highest DNA binding constant observed in the present work. A similar behavior was observed for *m*-chloro derivative **10**, Group C (K_b_ = 1.31(±0.08) × 10^6^ M^−1^). As concerning the effect of a halogen atom as the substituent, *p*-Br-derivative **12** bears the highest K_b_ value (=9.13(±0.15) × 10^5^ M^−1^).

**Figure 4:**
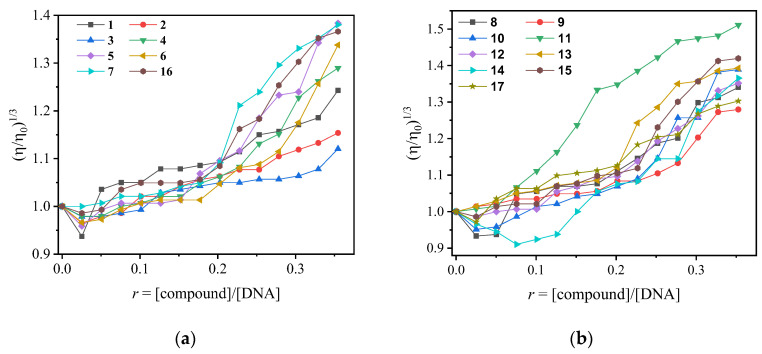
Relative viscosity (η/η_0_)^1/3^ of CT DNA (0.1 mM) in buffer solution (150 mM NaCl and 15 mM trisodium citrate at pH 7.0) in the presence of increasing amounts of compounds **1**–**17** (*r* = [compound]/[DNA] = 0–0.36). (**a**) Groups A and B; (**b**) Groups C and D.

Any changes in the DNA structure upon the addition of a studied compound have also been monitored via viscosity experiments (SI, Part S.2.2) that provide information about the mode of interaction due to their sensitivity to the relative DNA length changes (L/Lo) [80]. More broadly speaking, when a compound intercalates into DNA, the distance between the DNA base pairs increases at the intercalation site to facilitate the insertion of the hosted compound. Thus, the relative DNA length increases, leading to an increase in DNA viscosity, whose value is often proportional to the strength of the interaction [81]. In the case of non-classical intercalation (i.e., electrostatic interaction or groove-binding), the relative DNA length suffers rather than a slight shortening, and accordingly, a slight decrease in the DNA viscosity may be induced [81]. Within this context, the viscosity of a CT DNA solution (0.1 mM) was monitored upon the addition of increasing amounts of the compounds (up to the value of *r* = 0.36, Figure 4). Initially and up to the r-value of 0.1, the viscosity of the CT DNA solution remains practically stable, suggesting an external interaction with the compounds (obviously groove-binding). For r-values above 0.1, the observed increase in DNA viscosity could be attributed to an intercalative interaction [60,61,62,63]. 

**Figure 5:**
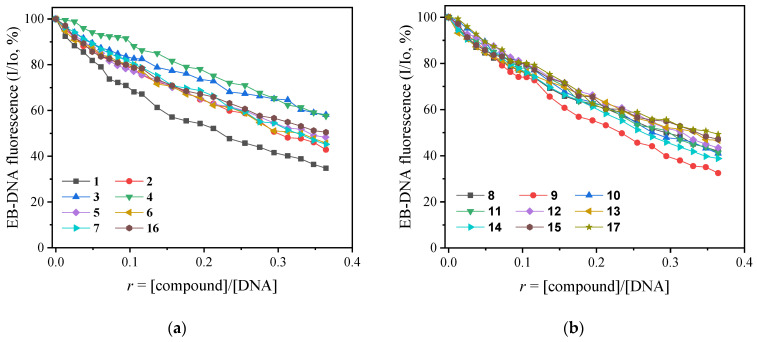
Plot of EB–DNA relative fluorescence emission intensity at λ_emission_ = 592 nm (%) versus *r* (*r* = [compound]/[DNA]) in the presence of compounds **1**–**17**. (**a**) Groups A and B; (**b**) Groups C and D; (up to 34.8 % of the initial EB–DNA fluorescence emission intensity for **1**, 42.9 % for **2**, 59.2 % for **3**, 57.6 % for **4**, 48.4% for **5**, 45.8 % for **6**, 45.2 % for **7**, 42.0 % for **8**, 32.5 % for **9**, 41.0 % for **10**, 41.3 % for **11**, 43.4 % for **12**, 46.7 % for **13**, 38.8 % for **14**, 47.1 % for **15**, 50.5 % for **16**, 49.3 % for **17**).

EB is a fluorescent dye that intercalates DNA and forms an adduct with an intense fluorescent emission band at 592–593 nm, when excited at 540 nm [82]. When a compound that intercalates into DNA equally or more strongly than EB is added to the EB-DNA solution, changes in the EB-DNA emission band may be observed and are often monitored to examine the competition of the compound with EB for the DNA intercalation site [82]. Thus, the fluorescence emission spectra of 1 h pretreated EB-DNA ([EB] = 20 µM, [DNA] = 26 µM) were recorded in the presence of increasing amounts of the compounds (see the case of compound **9** in Figure 3b) and a significant decrease in the fluorescence emission band of EB-DNA at 592 nm (up to 67.5% for compound **9**, Figure 5b, Table 2) revealed that the compounds are able to displace EB for the EB–DNA adduct. Thus, an intercalative mode of interaction of the complexes with CT DNA can be indirectly proposed [83].

The Stern–Volmer (K_SV_) constants (Table 2) of the complexes were calculated with the Stern–Volmer equation (SI, Part S.2.3, Equation (2)) and the corresponding S–V plots (Figure Part S.4). K_SV_ are relatively high, compounds **4** and **8** exhibit the highest values (8.40 × 10^4^ and 8.51 × 10^4^ M^−1^, respectively), suggesting a tight binding to DNA. In addition, the EB-DNA quenching constants (k_q_) of the compounds (Table 2) were calculated with Equation (3) (SI, Part S.2.3) (considering τ_o_ = 23 ns as the fluorescence lifetime value) [84] and are higher than the value 10^10^ M^−1^s^−1^ [83]. Therefore, a static quenching mechanism may be proposed for the quenching of the fluorescence induced by the compounds [82], suggesting subsequently the interaction of the compounds with the fluorophore.

### 2.3. DNA Interactions of Arylazo Sulfones with Plasmid DNA pBluescript SK II 

#### 2.3.1. DNA Cleavage Experiments 

All compounds, at DMSO solutions ≤10%, were incubated with plasmid DNA pBluescript SK II (500 ng). To confirm the stability of the examined compounds in DMSO, NMR experiments in DMSO-*d*_6_ and other deuterated media (DMSO-*d*_6_/D_2_O and CD_3_OD, t = 48 h) were carried out. The compounds were found to be stable in DMSO, the solvent used for their storage (SI, Part S.5). It should be noted that the samples immediately after their preparation in DMSO were kept at 4 °C, in the fridge. 

**Figure 6:**
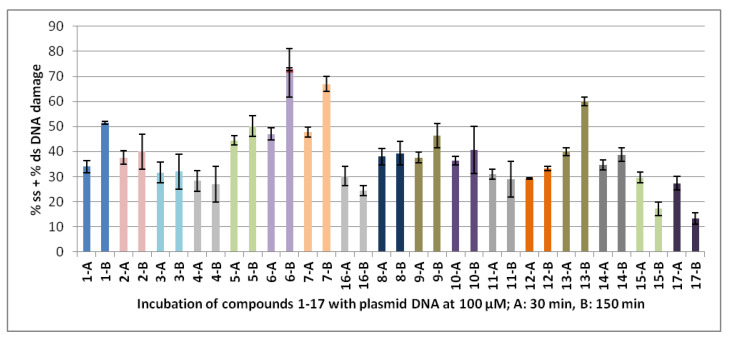
Plots of DNA cleavage of compounds **1**–**17** in dark. Explanation of the horizontal axis codes: **1-A**: The first number corresponds to the number of the compound in the text (compound **1** in the example). **A** means incubation of the compound with plasmid DNA for 30 min and concentration 100 μM. **B** means incubation of the compound with plasmid DNA for 150 min and concentration 100 μM. Pictures of the agarose gel electrophoresis of representative experiment of each compound under conditions **A** and **B** are given in SI, Part S.6.1. ds % is given always in red and on the top of the ss % plot.

The chosen concentration for the experiments in the dark was 100 μM, and the estimated incubation time was 30 and 150 min. According to the protocol used, the compounds are incubated for 30 min before irradiation, and then the irradiation lasts for two hours. As can be seen in Figure 6 (and S.I. Part S.6.1), incubation of the compounds in the dark for 30 and 150 min (**A** and **B**, respectively, for plots with the same color, in Figure 6), most of the compounds show their cleavage activity within the first 30 min. A slight increase over time has been observed for only a few compounds (e.g., **1**, **6**, **7**, **9**, and **13**). 

Having completed the control experiments in the dark, we proceeded to the irradiations of all compounds at 100 μM.

#### 2.3.2. DNA Photo-Cleavage Experiments

**Figure 7:**
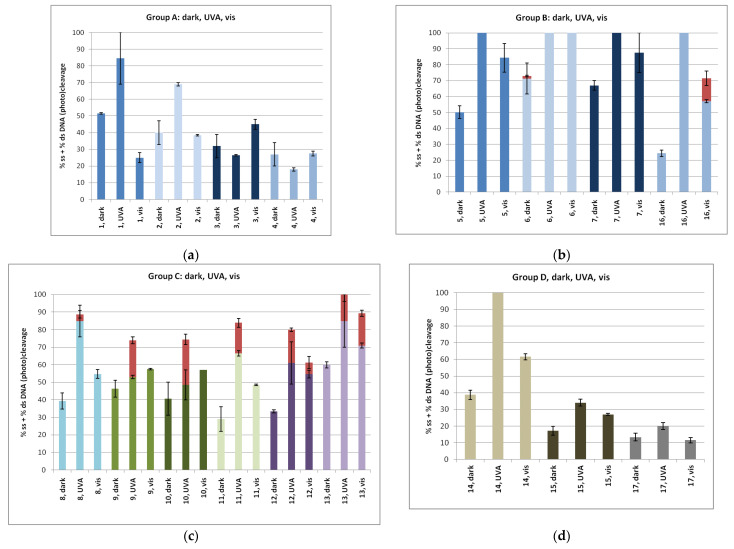
Plots of DNA cleavage and photocleavage for (**a**) Group A, (**b**) Group B, (**c**) Group C, and (**d**) Group D. For each compound, all three experiments at concentration 100 μM are depicted in the order: dark, UV-A, visible. Pictures of the agarose gel electrophoresis of representative experiment of each compound under UV-A and visible irradiation are given in SI, Part S.6.2. ds % is given always in red and on the top of the ss % plot.

All compounds at a concentration of 100 μΜ were mixed with pBluescript SK II, incubated for 30 min, and then irradiated for 120 min, either at 365 nm or with visible light [Figure 7a–d; each set of three same-colored plots indicates the average number of the % ss and % ds (photo)cleavage; the latter is always depicted on the top of the ss plot and in red]. The second column of each triad affords the result of the UV-A irradiation on each compound of the group, and the third column is the visible light irradiation result. The first column was added for comparison of the effect in dark (Figure 6 under the same concentrations and irradiation time; pictures of the agarose gel electrophoresis of representative experiment of each compound under UV-A and under visible irradiation are given in SI, Part S.6.2)]. UV-A irradiation caused obviously a stronger DNA photocleavage than upon visible light for most of the compounds (Group A, compounds **1** and **2**; Group B, compounds **5**, **7**, and **16**; Groups C and D, all compounds). This is probably due to the significant absorption of the compounds in the UV-A region (SI, Part S.7) and the higher energy offered by UV-A irradiation compared to visible light. Nitro derivatives proved to be very effective, and a lower concentration had to be examined. Gratifyingly, particularly for compound **5**, it was found very active even at a concentration of 25 μM (SI, Part S.6.3) and led to a cleavage of 50% of the plasmid between 10 and 25 μM.

Mechanistic studies for compound **1** under UV-A showed that the DNA photocleavage has been reduced both under argon and under air in the presence of scavengers of singlet oxygen (such as histidine and NaN_3_) and in the presence of hydroxyl radical scavengers (DMSO and KI), indicating that, among different ROS, singlet oxygen is effectively formed (SI, Part S.8). Similar behavior towards various scavengers has been observed for compound **5**. In visible light, the singlet oxygen formation was obvious. 

### 2.4. Molecular Docking “In Silico” Calculations of DNA/Arylazo Sulfones

**Figure 8:**
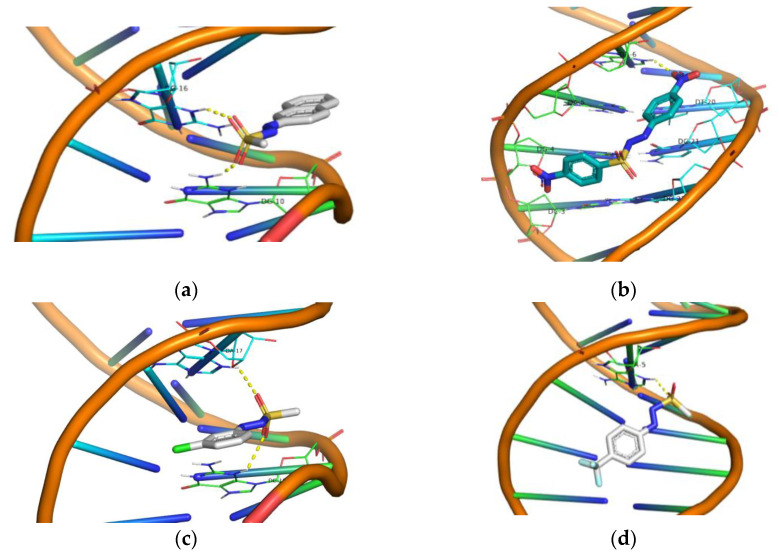
3D structures of the polar contacts of selected compounds of each group. (**a**) Compound **1** of Group A; (**b**) compound **16** of Group B; (**c**) compound **10** of Group C; (**d**) compound **14** of Group D. See Figures S.9.1–S.9.4 for further details.

Molecular docking studies for all derivatives **1**–**17** were performed, utilizing the AutoDock Vina program. The scope was to identify the polar contacts and calculate the energy of their DNA binding. In Table 3, all calculated energy binding values as well as DNA base interactions are provided. We may see that in Group A naphthyl derivative (**1**) shows the highest binding energy, meaning that probably the planarity offered by the extra aromatic ring increases stacking with DNA base pairs. Additionally, polar interactions are developed with the participation of both oxygen atoms that constitute the sulfonyl moiety (Figure 8a), whereas in compounds **2**–**4**, the polar interaction involves only one oxygen atom (SI: Part S.9.1, Group A).

Compound **4** exhibits also comparable energy binding with the naphthylazo sulfone **1**, and it seems to give superior values in UV binding experiments (K_b_, Table 1) and in competitive studies with EB (K_sv_ and K_q_, Table 2). In viscosity experiments up to *r* ~ 0.25, compound **1** shows better interaction that is inversed in favor of compound **4** at higher *r* values (Figure 4). In cleavage and photocleavage experiments, compound **1** was superior to the remaining derivatives (Figure 7a). 

In Group B, compound **16** shows the highest binding energy, with *p*-, *m*- and *o*- derivatives showing quite similar values, albeit lower than **16** (Table 3, Figure 8b, SI: Part S.9.2, Group B). We may observe, however, that the lack of the *p*-nitro-phenylsulfonyl aromatic ring in compounds **5**–**7** allows them to possess more polar contacts. This is more obvious for the *m*- and *o*- derivatives (**6** and **7**, respectively) where both nitro as well as sulfonyl moieties develop polar contacts to DNA (SI: Part S.9.2, Group B). In all DNA affinity calculations using spectroscopic techniques, compounds **5** and **6** were found to have a stronger effect than compounds **7** and **16,** with the exception of the viscosity experiments, in which compounds **5**, **7**, and **16** exhibited almost equally high values up to *r* ~ 0.36. As for DNA photocleavage, it is not safe to arrive at conclusions due to the high reactivity of the compounds at the concentrations used in the experiments (Figure 7b). DNA photocleavage is a complex phenomenon that requires not only a good affinity to DNA for the photo-derived radicals to attack DNA but also the generation of those radicals, which needs an efficient intersystem crossing of the photosensitizer to its triplet state, which is a physico-chemical property of each individual compound [85].

The compounds of Group C seem to exhibit quite similar energy bindings (Table 3, Figure 8c, SI: Part S.9.3, Group C) and this is observed in the DNA cleavage and photocleavage experiments, with the exception of compound **13** (Figure 7c). Finally, in Group D, it seems that polar interactions are important for DNA cleavage and photocleavage since those that lack such interactions show very poor activity (Table 3, Figure 8d, Figure 7d and Figure SI: Part S.9.4, Group D). However, their spectroscopically calculated bindings to CT-DNA were comparable to most of the compounds. 

### 2.5. Cell Culture Experiments of Arylazo Sulfones with Melanoma Cell Lines

**Figure 9:**
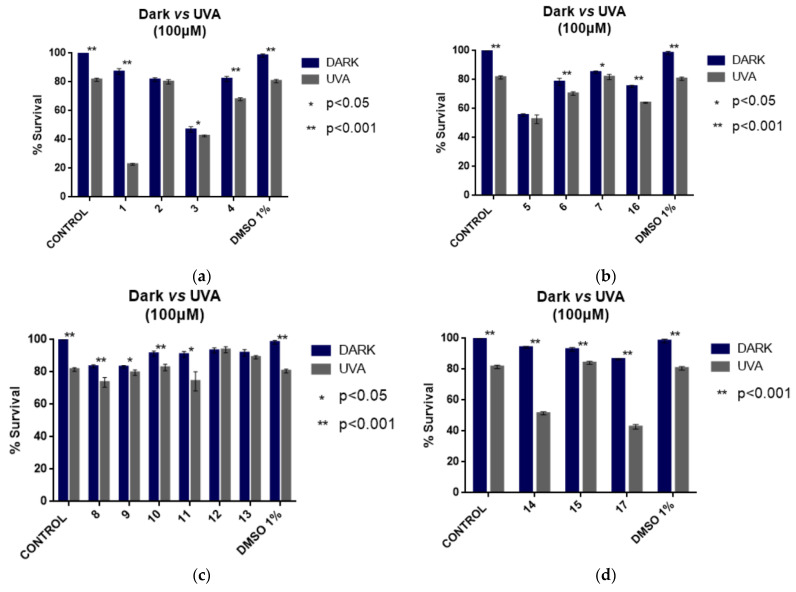
A375 melanoma cell culture viability experiments. Effectiveness of compounds **1**–**17** in dark and under UV-A irradiation; (**a**) Group A; (**b**) Group B; (**c**) Group C; (**d**) Group D.

Highly malignant melanoma cell lines have been used for cell culture experiments. Control experiments have been performed in dark and under irradiation of the cells without the presence of any arylazo sulfone. A second set of control experiments provided information about the effect of the presence of 1% DMSO in the culture media, which was the final concentration of DMSO in the solutions with the dissolved tested compounds. As shown, the presence of 1% DMSO was well tolerated by the cells (Figure 9). For the photodamage experiments, the cells were incubated individually with each of the seventeen compounds (100 μM and 50 μM) for 1 h and then irradiated under UV-A light for 1 h. Cell culture media with the compounds were then removed, and viability was measured after 24 h. 

In Group A, it was observed that compound **1** induced satisfactory photodamage, causing death in about 80% of the cells. By taking into account the “phototoxicity” caused by the 1% DMSO alone (after normalization), the death of the cells is ~60% (Figure 9a). This is the first indication that naphthyl-azo methylsulfone (**1**) is a “true” photo-disrupting compound that is activated only in the presence of irradiation. Compound **3** exhibited the same percentage of dead cells regardless of the use of irradiation, reaching its IC_50_ at 100 μM (Figure 9a). However, derivatives **2** and **4** were able to cause some minor cell damage when used in the dark, with the latter responding to irradiation, albeit to a lesser extent than compound **1**. When the concentration of the compounds was reduced to 50 μM, compound **1** continued to cause the same high rate of cell death, but the activity of derivative **3** was reduced, whereas that of derivatives **2** and **4** remained the same (SI: Part S.10.1, Group A). 

As far as the nitro group-containing derivatives (Group B) are concerned, compound **5** reached its IC_50_ concentration at 100 μM. Irradiation did not cause any additional damage. Under the dark, the effect of compounds **6**, **7**, and **16** was weak; however, irradiation improved the effect on derivative **16**. Lower compound concentrations reduced their effect in the dark while retaining or increasing the photochemical effect (Figure 9b, SI: Part S.10.2, Group B). The effect of all halogenated compounds of Group C on melanoma cells was weak; however, here again, lowering the concentration favored the photochemically over the chemically (in the dark) caused death of the cells (Figure 9c, SI: Part S.10.3, Group C).

It was very interesting to note that among compounds **14**, **15**, and **17** of Group D, compound **14** and derivative **17** showed equal activity, with both compounds reaching their IC_50_ at this concentration. Compound **14**, apart from the exhibited photo-disruptive activity on melanoma cells, showed the highest DNA-binding constant (K_b_, Table 1), EB-DNA Stern–Volmer and EB-DNA quenching constants (K_SV_, k_q_, Table 2), DNA cleavage and photocleavage (Figure 8d) and “in silico” calculated energy bindings (Table 3). No activity has been observed in the dark. Photo-reactivity was decreased when the concentration was lowered to 50 μM (Figure 9d, SI: Part S.10.4, Group D). Compound **15**, which contained sulfur stripped of oxygen atoms, exhibited no activity, not only in cells but also towards plasmid DNA (Figure 7). Even though more experimentation is required, it seems that the azosulfone moiety is important for showing the examined biological activities. The exchange of sulfur to nitrogen showed that the derived compounds should be considered as a different class of possible photosensitizers, as experiments with plasmid DNA and most importantly cell culture experiments indicated most probably a different mechanism with the cells which are in due course.

**Figure 10:**
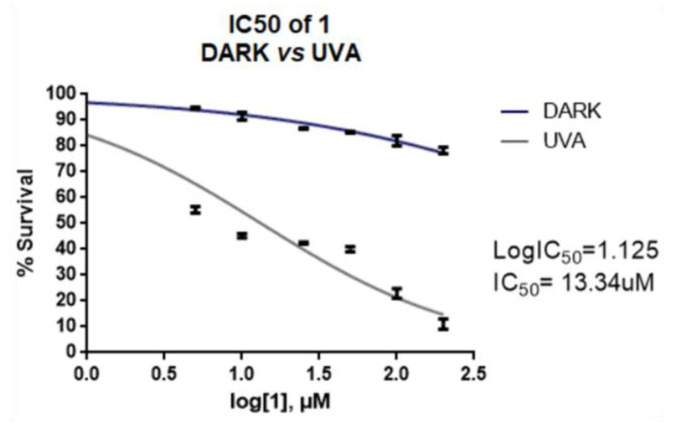
IC_50_ determination for the effect of compound **1** on melanoma cells.

Concluding the results of the cell cultures with melanoma cells, it was found that compounds **1**, **14**, and **17** exhibited only photochemical activity, with the two last compounds showing an IC_50_ of 100 μM and derivatives **3** and **5** having a chemical activity with the same IC_50_. Derivative **1**, however, was superior to all, and therefore for its IC_50_ to be determined, A365 melanoma cells were incubated with six different concentrations of **1** (0, 5, 10, 25, 50, 100, 200 μM) following the same procedure, in the dark and under UV-A irradiation (Figure 10).

The results were very encouraging. Cells’ viability without irradiation was greater than 50% even at higher concentrations than 200 μM, whereas the IC_50_ under UV-A irradiation was 13.34 μM.

### 2.6. Cell Culture Experiments of the Photoactive Arylazo Sulfones ***1***, ***14*** and ***17*** with Non-Cancer Cell Lines

**Figure 11:**
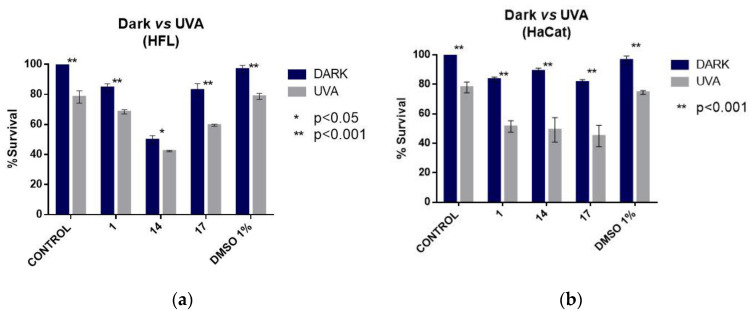
Non-cancer cell lines experiments with compounds **1**, **14**, and **17** in dark and under UVA; (**a**) Effect on HFL1 fibroblast cell line; (**b**) Effect on human immortalized keratinocytes.

In order to perform a control experiment with non-cancer cells, two different cell lines were used; HFL1, a fibroblast cell line that was isolated from the lung of a white, normal embryo, and HaCaT cell lines, human immortalized keratinocytes (Figure 11a,b, respectively). Cells were incubated with 13.4 μM of compound **1** and 100 μM of compounds **14** and **17**. The experimental procedure was the same as the procedure followed for A375 cell lines. As can be seen in Figure 11, in these preliminary results, compounds **1** and **17** were less toxic for the fibroblast cell line than the keratinocyte ones. Nevertheless, the photodamage in keratinocytes was comparable to that in melanoma cells (Figure 9a and Figure 10 for compound **1** and Figure 9d for compounds **14** and **17**). The damage was attributed to the UV-A irradiation in combination with the compound. The compounds themselves were found non-toxic for the cells in dark. Thus, it seems that the advantage of the spatial and temporal control driven by light, in conjunction with the novelty of the arylazosulfone scaffold, may be useful for the development of phototoxic pharmaceutics.

## 3. Materials and Methods

All commercially available reagent-grade chemicals and solvents were used without further purification. Trisodium citrate, NaCl, CT DNA, and EB were purchased from Sigma-Aldrich Co., and all solvents were from Chemlab. DNA stock solution was prepared by dilution of CT DNA to buffer (containing 150 mM NaCl and 15 mM trisodium citrate at pH 7.0) followed by exhaustive stirring at 4 °C for 3 days and kept at 4 °C for no longer than a week. The stock solution of CT DNA gave a ratio of UV absorbance at 260 and 280 nm (A_260_/A_280_) of ~1.90, indicating that the DNA was sufficiently free of protein contamination [86]. The DNA concentration per nucleotide was determined by the UV absorbance at 260 nm after 1:20 dilution using ε = 6600 M^−1^ cm^−1^ [87]. The supercoiled plasmid pBluescript SK II was synthesized and tested not to contain nicked and/or linear strands. All samples containing pBluescript SK II were irradiated at pH 6.8 with Philips 2 × 9W/10/2P UV-A lamps at 365 nm or white light OSRAM DULUX S BLUE. NMR spectra were recorded on an Agilent 500/54 (500 MHz for ^1^H) (Agilent Technologies, Santa Clara, CA, USA) and on a Bruker (300 MHz for ^1^H) spectrometer using DMSO-*d*_6_, D_2_O, CDCl_3_, and CD_3_OD as solvents. UV–vis spectra were recorded on a Hitachi U–2001 dual-beam spectrophotometer (Hitachi, Tokyo, Japan). Viscosity experiments were carried out using an ALPHA L Fungilab rotational viscometer (Fungilab, Barcelona, Spain) equipped with an 18 mL LCP spindle and the measurements were performed at 100 rpm. Fluorescence spectra were recorded in solution on a Hitachi F-7000 fluorescence spectrophotometer (Hitachi, Tokyo, Japan). 

Arylazo sulfones **1**–**14** were prepared from the corresponding diazonium salts by following a known procedure [64]. Spectroscopic data for compounds **1, 11, 12** [88], **2**–**4** [40] **5, 6**–**9, 14** [64], **10** [67], **13** [89] were in accordance with the literature. Compound **17** was synthesized from 4-bromoaniline by following a known procedure [90]. Spectroscopic data for **17** were in accordance with the literature [91].

Synthesis of 4-(((4-chlorophenyl)thio)diazenyl)benzonitrile (**15**).

Compound **15** was prepared by adapting a known procedure [92]. 4-aminobenzonitrile (11.4 mol) was suspended in MeCN:H_2_O 2:1 (5 mL), and the resulting mixture was cooled to 0 °C. Conc. HCl (3.8 mL) was then added, and the so-obtained mixture was further cooled to −5 °C. A solution of NaNO_2_ (17 mmol) in water (5 mL) was added dropwise, and the mixture was treated with sodium acetate (25% *w/w*) until pH = 5. 4-chlorothiophenol (11.4 mmol) in ethanol (20 mL) was then added dropwise, and the resulting orange precipitate was collected by filtration and purified by recrystallization from ethanol, to afford 1.81 g of **15** (orange solid, 58% yield, mp (dec.): 111.3–112 °C). 

**15**. ^1^H NMR (300 MHz, CDCl_3_) δ: 7.47–7.50 (d, 2H, *J* = 6Hz), 7.58–7.63 (m, 4H), 7.71–7.74 (d, 2H, *J* = 6 Hz). ^13^C NMR (75 MHz, CDCl_3_) δ: 113.5, 118.4, 122.4, 129.7, 131.5, 132.9, 133.4, 136.4, 153.5. Anal. Calcd for C_13_H_8_ClN_3_S: C, 57.04; H, 2.95; N, 15.35. Found: C, 57.1; H, 3.0; N, 15.2.

Synthesis of 1-(4-nitrophenyl)-2-((4-nitrophenyl)sulfonyl)diazene (**16**).

Compound **16** was prepared by adapting a known procedure [63]. 4-nitrophenyldiazonium tetrafluoroborate (1.89 g, 8 mmol) and sodium 4-nitrobenzensulfinate [93] (1.77 g. 8.5 mmol) were suspended in CH_2_Cl_2_ (20 mL). The resulting mixture was stirred overnight, then filtered, and the obtained solution was evaporated to give a dark yellow residue, that was purified by dissolving in CH_2_Cl_2_ and precipitated by adding cold n-hexane, to afford 336 mg of **16** (yellow solid, 12.5% yield, mp (dec.): 129–130 °C. 

**16.** ^1^H NMR (300 MHz, CD_3_COCD_3_) δ: 8.60 (d, *J* = 8.9 Hz, 2H), 8.49 (d, *J* = 9.0 Hz, 2H), 8.33 (d, *J* = 8.9 Hz, 2H), 8.11 (d, *J* = 9.0 Hz, 2H). ^13^C NMR (75 MHz, CD_3_COCD_3_) δ: 153.3, 153.2, 152.7, 139.1, 133.6, 126.6, 126.5, 125.8. Anal. Calcd for C_12_H_8_N_4_O_6_S: C, 42.86; H, 2.40; N, 16.66. Found: C, 43.0; H, 2.5; N, 16.5.

### 3.1. Interaction with CT DNA

The interaction of the compounds with CT DNA was evaluated in vitro using their solutions in DMSO (1 mM) due to their low solubility in water. These studies were performed in the presence of aqueous buffer solutions, where the mixing of each solution never exceeded 5% DMSO (*v/v*) in the final solution. Control experiments were undertaken to assess the effect of DMSO on the data, and no changes were observed in the spectra of CT DNA. The interaction of the compounds with CT DNA was investigated by UV–vis spectroscopy and viscosity measurements, and the evaluation of their EB-displacing ability was studied by fluorescence emission spectroscopy. Detailed procedures and equations regarding the in vitro study of the interaction of the compounds with CT DNA are given in the Supporting Information File (SI, Parts S.2–S.4).

### 3.2. DNA Cleavage and Photo-Cleavage Experiments

Compounds **1**–**17** were individually incubated with plasmid DNA at the desired concentration in Eppendorf vials and/or irradiated with UV-A or visible light (365 nm-18 W, or white light 400–700 nm-18 W) and in 10 cm distance under aerobic conditions at room temperature for 2 h. Conditions of the photobiological reaction and gel electrophoresis, quantification of DNA cleaving activity, and calculation of ss% and ds% damage protocols have been described previously [60]. All experiments were performed at least twice. 

### 3.3. Molecular Docking Studies

Organic compounds were fully optimized at the B3LYP/6-31g* level of theory with the LanL2DZ basis set for iodine in the case of compound **13** as implemented in the Gaussian 09 [94] suite of programs (Revision B.01). The crystal data of the B-DNA dodecamer d(CGCGAATTCGCG)2 (PDB 1D:1BNA) were downloaded from the Protein Data Bank [95]. The docking analysis was performed using the AutoDock Vina program [96]. The DNA was adapted for docking by removing water molecules and polar hydrogens, and Gasteiger charges were added by Autodock 4.2 Tools (ADT) before performing docking calculations. A grid box with a size of 60 × 80 × 114 with 0.375 Å spacing was used to encompass the whole DNA. The rigid docking protocol and 100 runs of the Lamarckian genetic algorithm for searching ligand conformations were performed. PyMOL [97] was used for the representation of the docking results and interactions between DNA and compounds.

### 3.4. Cell Culture Experiments

A375 (CRL-1619^TM^) cell line was used to test the cytotoxic effect of the compounds [76]. Cells were cultured under aseptic conditions using DMEM basal medium (31885-023; Gibco, MD, USA) supplemented with 10% fetal bovine serum (FB1000/500, Biosera, London, UK), 100 units/ml penicillin, 100 ug/mL streptomycin (15140-122, Gibco), and 2 mM L-glutamine (25030; Gibco). The cell line was maintained at standard conditions (37 °C, 5% CO_2_) in a humidified atmosphere and was used at 70–90% confluency. Five thousand cells were seeded per well. A UV-A lamp was placed 10 cm over the 96-well plate. After 1 h incubation with 50 μM and 100 μM of each compound, 1 h irradiation with UV-A (365 nm) followed. Then, compounds were removed, and a cytotoxicity assay was performed 24 h later. Resazurin Cell Viability Assay (CA035, Canvax, Valladolid, Spain) was used for fluorescence measurements according to the manufacturer’s guidelines. Essentially, a non-irradiated 96-well plate was used as a control, under the same conditions. Incubation with 10% resazurin (7 h) was followed by fluorescence measurement at λ_em_ = 590 nm and λ_ex_ = 530/560 nm in a VarioSkan lux reader (Thermo, MT, USA).

HFL1, a fibroblast cell line, was isolated from the lung of a white, normal embryo, as were HaCaT cell lines, human immortalized keratinocytes. Cells were cultured under aseptic conditions using DMEM basal medium (31885-023; Gibco) supplemented with 10% fetal bovine serum (FB1000/500, Biosera, UK), 100 units/ml penicillin, 100 ug/mL streptomycin (15140-122, Gibco), and 2 mM L-glutamine (25030; Gibco). Cell lines were maintained at standard conditions (37 °C, 5% CO_2_) in a humidified atmosphere and were used at 70–90% confluency. Five thousand cells were seeded per well. Cells were incubated with 13.4 μM of compound **1** and 100 μM of compounds **14** and **17**. The experimental procedure was the same as the procedure that was followed for A375 cell lines.

## 4. Conclusions

A set of arylazosulfone derivatives has been synthesized, and their biological evaluation has been investigated in relation to their strong UV-A and visible light absorption and the lability of their N–S bond. Thus, their ability to photocleave DNA as well as their cytotoxic effect on the highly malignant melanoma cells A375 as well as on two non-cancer cell lines has been studied. 

The affinity of sulfones to calf-thymus DNA has been studied to prove their ability to interact with biological materials via polar contacts and Van der Waals forces. The interaction of compounds **1**–**17** with CT DNA revealed their tight binding to CT DNA via partial intercalation. It was found that the *m*-substitution (in the case of NO_2_- and Cl- derivatives) leads to higher DNA-binding constants. Molecular docking calculations have indicated moderate energy bindings and polar contacts for most of the compounds. 

Incubation of the compounds with plasmid DNA showed DNA cleavage for several derivatives, whereas application of light led to considerable DNA photocleavage, especially in the UV-A region. The derivatives that exhibited the best photocleavage activity were compounds **1**, **5**–**14**, and **16**, the vast majority of which had a nitro- or halogen-aromatic substituent. 

Cell cultures with the melanoma cells showed that derivatives **3** and **5** exhibited chemical activity with an IC_50_ of ~100 μM. On the other hand, compounds **1**, **14**, and **17** exhibited only photochemical activity with an IC_50_ of ~13, 100, and 100 μM, respectively. The same was evident for the two non-cancer cell lines. Derivatives **14** and **17**, and, most importantly, naphthyl derivative **1**, exerted a small effect in the dark on cells, but they killed them in concentrations as low as 100, 100, and ~13 μM, respectively. Thus, they might be lead compounds for the development of novel derivatives able to act under photodynamic effects and to be used in the development of phototoxic pharmaceuticals. 

## Figures and Tables

**Table 1:** Spectral features of the UV–vis spectra of compounds **1**–**17** upon addition of CT DNA. UV-band (λ, nm) (percentage of observed hyper-/hypo-chromism (ΔA/A_0_, %), blue-/red-shift of the λ_max_ (Δλ, nm)) and the corresponding DNA-binding constants (K_b_, M^−1^).

Groups	No of Compound	Band (nm) (ΔA/A_0_ (%) ^1^, Δλ (nm) ^2^)	K_b_ (M^−1^)
Group A	**1**	272 (+2,+1); 385 (+17, 0)	3.73(±0.24) × 10^5^
	**2**	300 (+2, +1)	1.12(±0.15) × 10^5^
	**3**	347 (−4, +1)	5.04(±0.16) × 10^5^
	**4**	387 (−68, +11); 489 (+54, +6)	6.08(±0.15) × 10^5^
Group B	**5**	288 (+6, −2); 359 (sh) ^3^ (−43, +20)	9.31(±0.18) × 10^3^
	**6**	269 (+8, +2)	2.87(±0.10) × 10^7^
	**7**	289 (+2, +3)	4.59(±0.10) × 10^5^
	**16**	274 (+6, +3)	1.05(±0.08) × 10^6^
Group C	**8**	301 (+1, +1)	2.67(±0.27) × 10^5^
	**9**	298 (+2, +0)	3.81(±0.35) × 10^5^
	**10**	290 (+8, −1)	1.31(±0.08) × 10^6^
	**11**	308 (+4, +1)	6.41(±0.32) × 10^5^
	**12**	312 (+0.5, +2)	9.13(±0.15) × 10^5^
	**13**	330 (+8, −1)	6.02(±0.44) × 10^5^
Group D	**14**	279 (+12, 0)	7.42(±0.10) × 10^5^
	**15**	280 (+23, +8)	1.02(±0.04) × 10^5^
	**17**	322 (−3, +0)	3.82(±0.30) × 10^5^

^1^ “+” denotes hyperchromism, “−“ denotes hypochromism; ^2^ “+” denotes red-shift, “−“ denotes blue-shift; ^3^ “sh” = shoulder.

**Table 2:** Data of the EB-DNA competitive studies of compounds **1**–**17**. Percentage of EB-DNA fluorescence quenching (ΔI/I_o_, %), EB-DNA Stern–Volmer constants (K_SV_, M^−1^), and EB-DNA quenching constants (k_q_, M^−1^s^−1^) for compounds **1**–**17**.

Groups	Compound	(∆I/I_o_, %)	K_SV_ (M^−1^)	k_q_, M^−1^ s^−1^
Group A	**1**	65.2	2.40(±0.03) × 10^4^	1.04(±0.01) × 10^12^
	**2**	57.1	3.51(±0.06) × 10^4^	1.53(±0.03) × 10^12^
	**3**	41.8	4.41(±0.08) × 10^4^	1.92(±0.03) × 10^12^
	**4**	42.4	8.51(±0.26) × 10^4^	3.70(±0.11) × 10^12^
Group B	**5**	51.6	3.58(±0.06) × 10^4^	1.56(±0.02) × 10^12^
	**6**	54.2	3.41(±0.07) × 10^4^	1.48(±0.03) × 10^12^
	**7**	54.8	1.31(±0.03) × 10^4^	5.70(±0.14) × 10^11^
	**16**	49.5	3.31(±0.05) × 10^4^	1.44(±0.02) × 10^12^
Group C	**8**	58.0	8.40(±0.13) × 10^4^	3.65(±0.05) × 10^12^
	**9**	67.5	5.88(±0.13) × 10^4^	2.56(±0.06) × 10^12^
	**10**	59	4.65(±0.12) × 10^4^	2.02(±0.05) × 10^12^
	**11**	58.7	4.20(±0.08) × 10^4^	1.83(±0.04) × 10^12^
	**12**	56.5	3.90(±0.11) × 10^4^	1.70(±0.05) × 10^12^
	**13**	53.3	3.69(±0.07) × 10^4^	1.60(±0.03) × 10^12^
Group D	**14**	61.2	4.37(±0.11) × 10^4^	1.90(±0.05) × 10^12^
	**15**	52.9	3.61(±0.08) × 10^4^	1.57(±0.03) × 10^12^
	**17**	50.7	3.77(±0.05) × 10^4^	1.64(±0.02) × 10^12^

**Table 3:** “In silico” energy binding calculations towards DNA of compounds **1**–**17**.

Groups	Compound	Energy (Kcal/mol)	Interactions (PyMol) Polar Contacts
Group A	**1**	−7.8	DG16, DG10
	**2**	−6.2	DA17
	**3**	−6.8	DG16, DA17
	**4**	−7.1	DG16, DA17
Group B	**5**	−7.2	DA17, DG10
	**6**	−7.3	DG10, DG12, DG14
	**7**	−7.3	DG10, DG14, DG16
	**16**	−8.9	DA16
Group C	**8**	−6.6	DA17, DG10
	**9**	−6.5	DG16, DA17
	**10**	−6.7	DG10, DA17
	**11**	−6.0	DG10, DA17
	**12**	−6.5	DG16, DA17
	**13**	−6.6	DG10, DA17
Group D	**14**	−7.3	DA5
	**15**	−7.3	No Polar Contacts
	**17**	−7.1	No Polar Contacts

## Data Availability

Not applicable.

## Supplementary Materials

The following supporting information can be downloaded at: https://www.mdpi.com/article/10.3390/ijms24031834/s1.
