# Supplementary material for: Effect of Arylazo Sulfones on DNA: Binding, Cleavage, Photocleavage, Molecular Docking Studies and Interaction with A375 Melanoma and Non-Cancer Cells
Source: Int J Mol Sci. 2023 Jan 17;24(3):1834. doi: 10.3390/ijms24031834 (PMC9915714; doi:10.3390/ijms24031834)
Supplement: Supplementary file 1 [file ijms-24-01834-s001.zip › ijms-2053083-supplementary.pdf]

Supporting Information

Effect of arylazo sulfones on DNA: Binding, Cleavage, Photo-cleavage and Molecular Docking Studies. Interaction with A375 melanoma and non-cancer cells

Chrysoula Mikra [1,‡], Achilleas Mitrakas[2,3,‡], Virginia Ghizzani[4], Katerina R. Katsani[5], Maria Koffa[2], Michael Koukourakis[3], George Psomas[6], Stefano Protti[4], Maurizio Fagnoni[4,*] and Konstantina C. Fylaktakidou[1,*]

1. Laboratory of Organic Chemistry, Faculty of Chemistry, Aristotle University of Thessaloniki, GR-54124 Thessaloniki, Greece; chrmikgeo@chem.auth.gr, kfylakta@chem.auth.gr
2. Laboratory of Cellular Biology and Cell Cycle, Molecular Biology and Genetics Department, Democritus University of Thrace, University Campus, Dragana, 68100 Alexandroupolis, Greece; amitrak@med.duth.gr, mkoffa@mbg.duth.gr
3. Department of Radiotherapy/Oncology, Democritus University of Thrace/University General Hospital of Alexandroupolis, Alexandroupolis, Greece; mkoukour@med.duth.gr
4. PhotoGreen Lab, Department of Chemistry, University of Pavia, V. Le Taramelli 12, Pavia 27100, Italy; virginia.ghizzani01@universitadipavia.it, stefano.protti@unipv.it, fagnoni@unipv.it
5. Laboratory of Biochemistry and Molecular Virology, Molecular Biology and Genetics Department, Democritus University of Thrace, Dragana, 68100 Alexandroupolis, Greece; kkatsani@mbg.duth.gr
6. Laboratory of Inorganic Chemistry, Faculty of Chemistry, Aristotle University of Thessaloniki, GR-54124 Thessaloniki, Greece; gepsomas@chem.auth.gr

‡ The two authors have contributed equally

* Correspondence: fagnoni@unipv.it (M.F.), kfylakta@chem.auth.gr K.C.F.

Table of contents

S.1	***Copies of NMR spectra of compounds 15 and 16***	S3
S.2<br>*S.2.1*<br>*S.2.2*<br>*S.2.3*<br>*S.2.4*	***Interaction with CT DNA***<br>*Binding study with CT DNA by UV-vis spectroscopy*<br>*CT DNA-binding studies by viscosity measurements*<br>*EB-displacement studies*<br>*References*	S7
S.3	***Plot of*** $\frac{[DNA]}{(\varepsilon_A - \varepsilon_f)}$ ***versus [DNA] of compounds 1-17***	S9
S.4	***Stern–Volmer quenching plot of EB–DNA fluorescence of compounds 1-17***	S12
S.5<br>*S.5.1*<br>*S.5.2*<br>*S.5.3*	***Copies of NMR spectra of 1, 5 and 8 in various solvents and time intervals***<br>*Compound **1** (DMSO-d_6, DMSO-d_6 & D_2O, CD_3OD)*<br>*Compound **5** (DMSO-d_6, DMSO-d_6 & D_2O, CD_3OD)*<br>*Compound **8** (DMSO-d_6, DMSO-d_6 & D_2O, CD_3OD)*	S15
S.6<br>*S.6.1*<br>*S.6.2*<br>*S.6.3*	***Gel electrophoresis pictures***<br>*Agarose gel electrophoresis of compounds **1-17** in dark*<br>*Agarose gel electrophoresis of compounds **1-17** under UVA and visible irradiation*<br>*Agarose gel electrophoresis of compound **5** in various concentrations under UVA irradiation*	S19
S.7	***Copies of UV-Vis spectra of compounds 1-17***	S20
S.8	***Gel electrophoresis analysis (mechanistic studies) of compounds 1 and 5***	S31
S.9	***In silico Molecular Dockings of compounds 1-17 with DNA***	S32
S.10	***Cell culture experiments of compounds 1-17 at concentration 50 μM***	S36

S.1 Copies of NMR spectra of compounds 15 and 16.

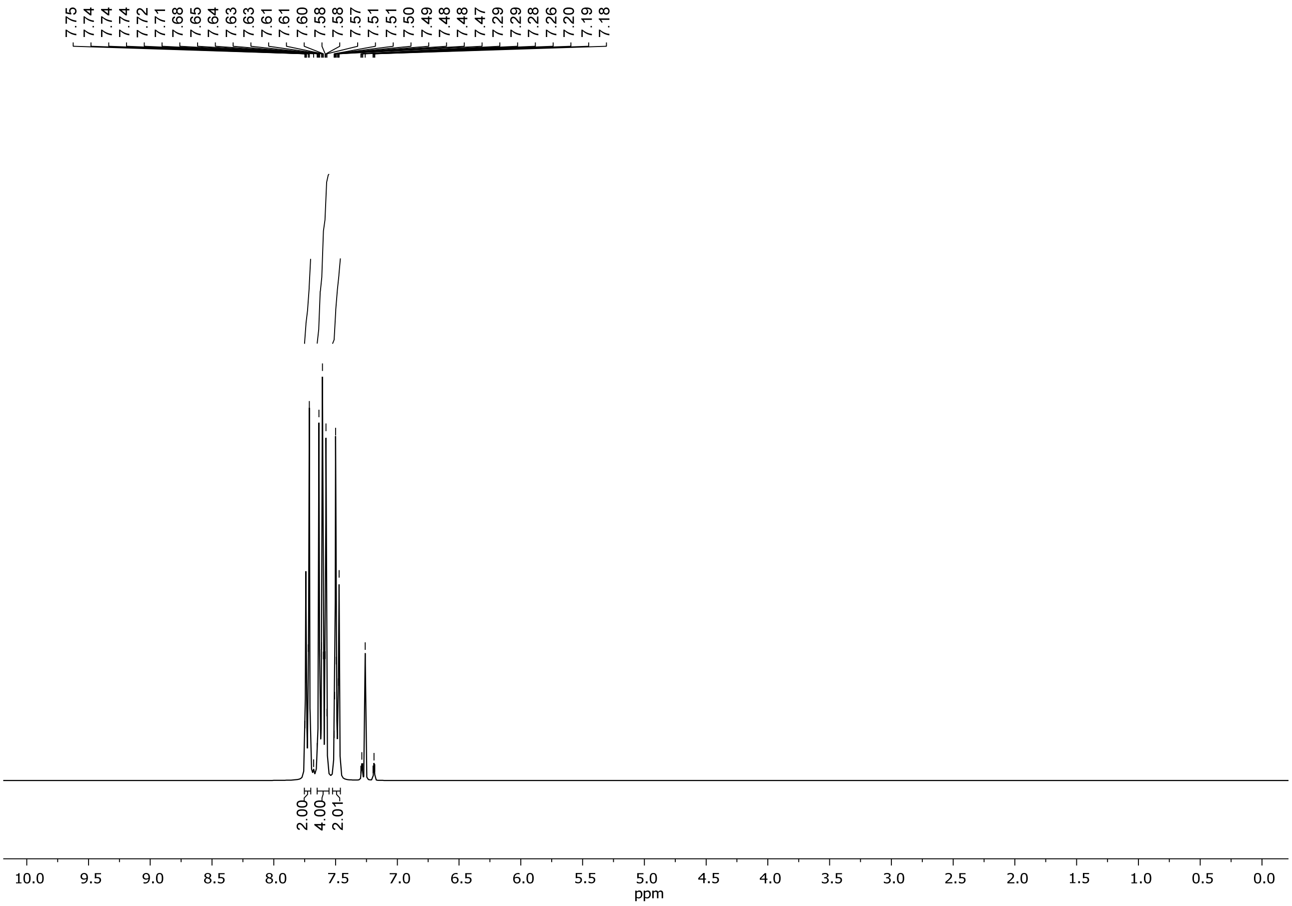

Figure part S.1.a. ^{1}H NMR spectra of compound **15** in $CDCl_3$ (300 MHz).

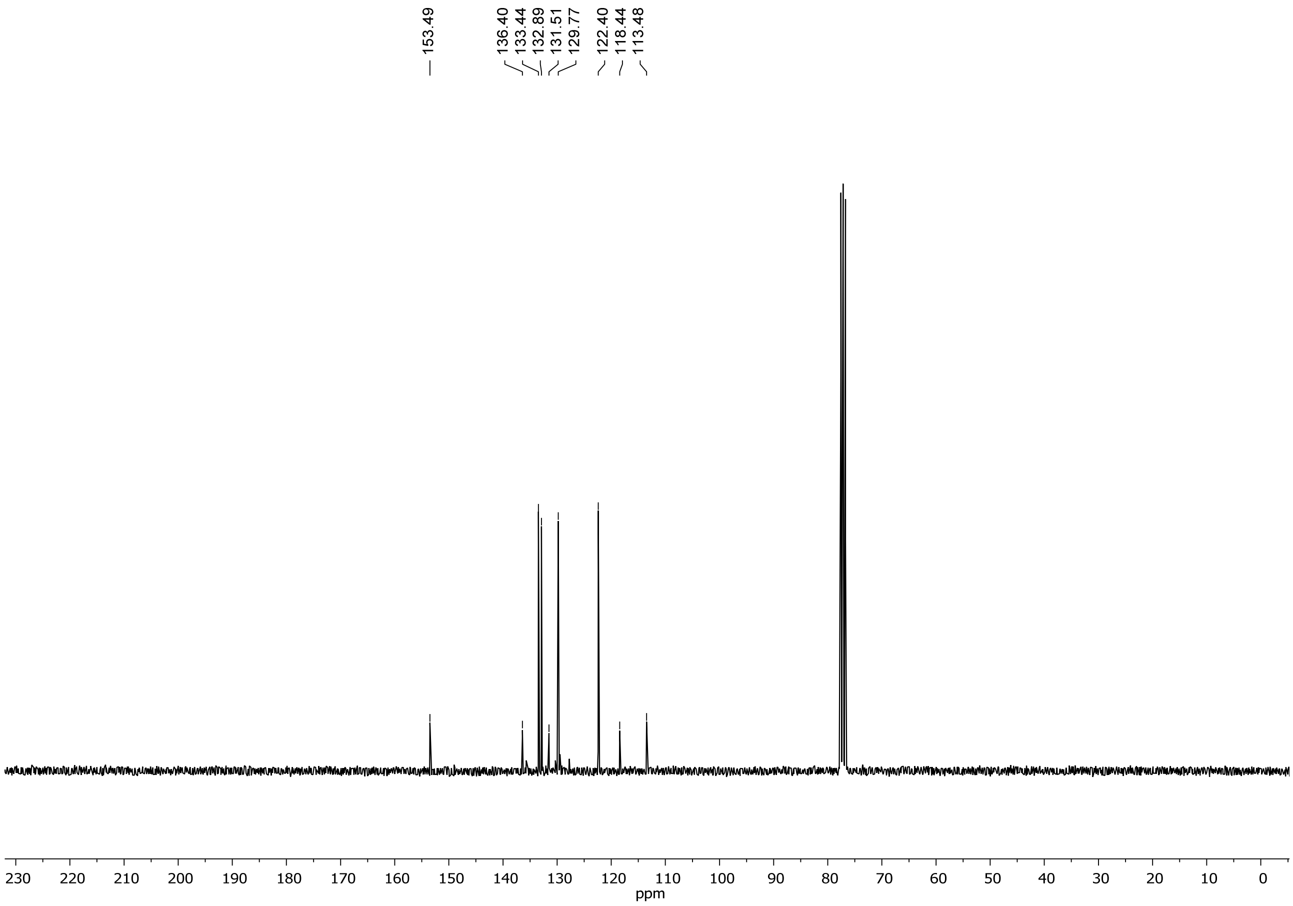

Figure part S.1.b. ^{13}C NMR spectra of compound **15** in $CDCl_3$ (75 MHz).

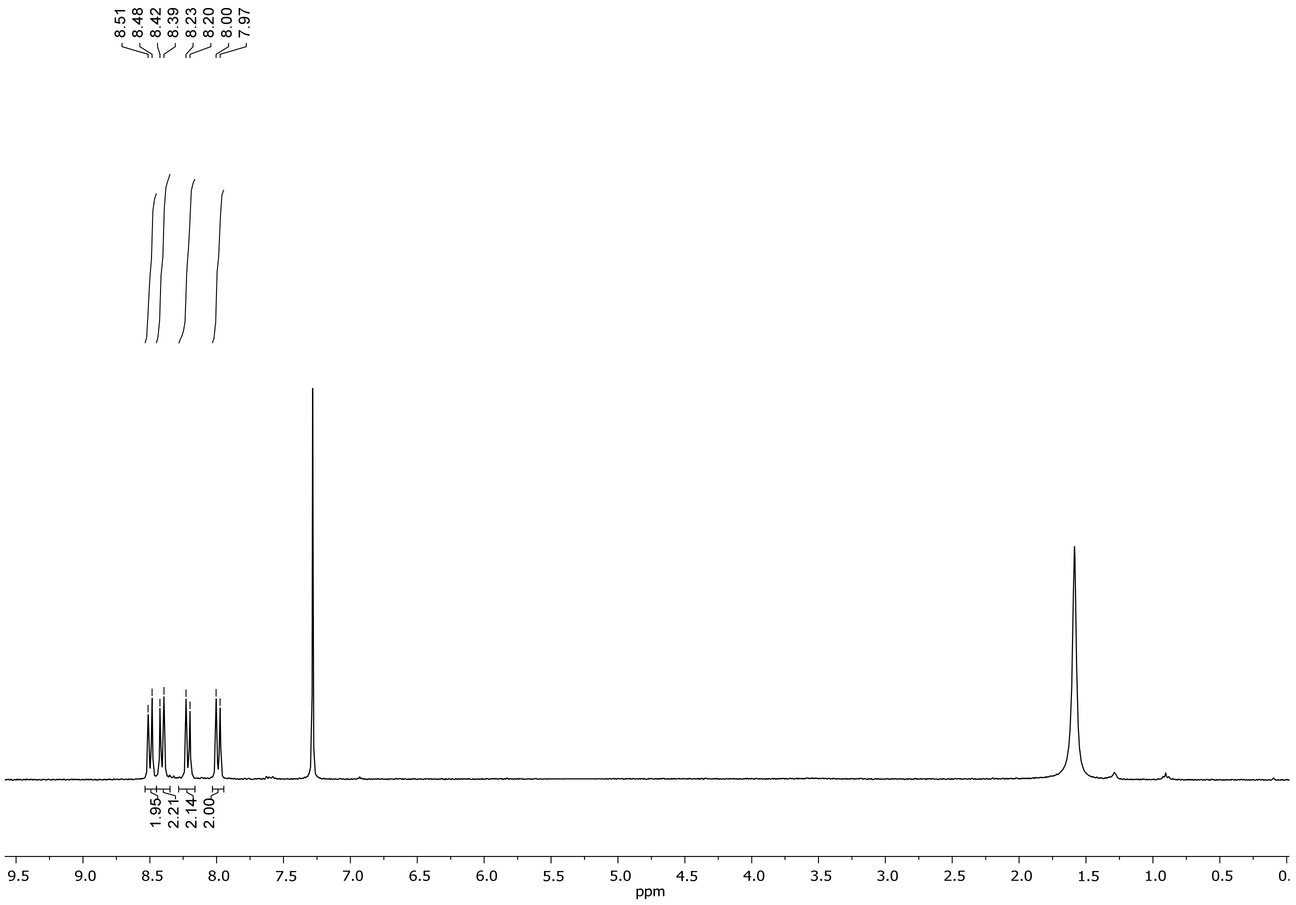

Figure part S.1.c. ^{1}H NMR spectra of compound **16** in $CDCl_3$ (300 MHz).

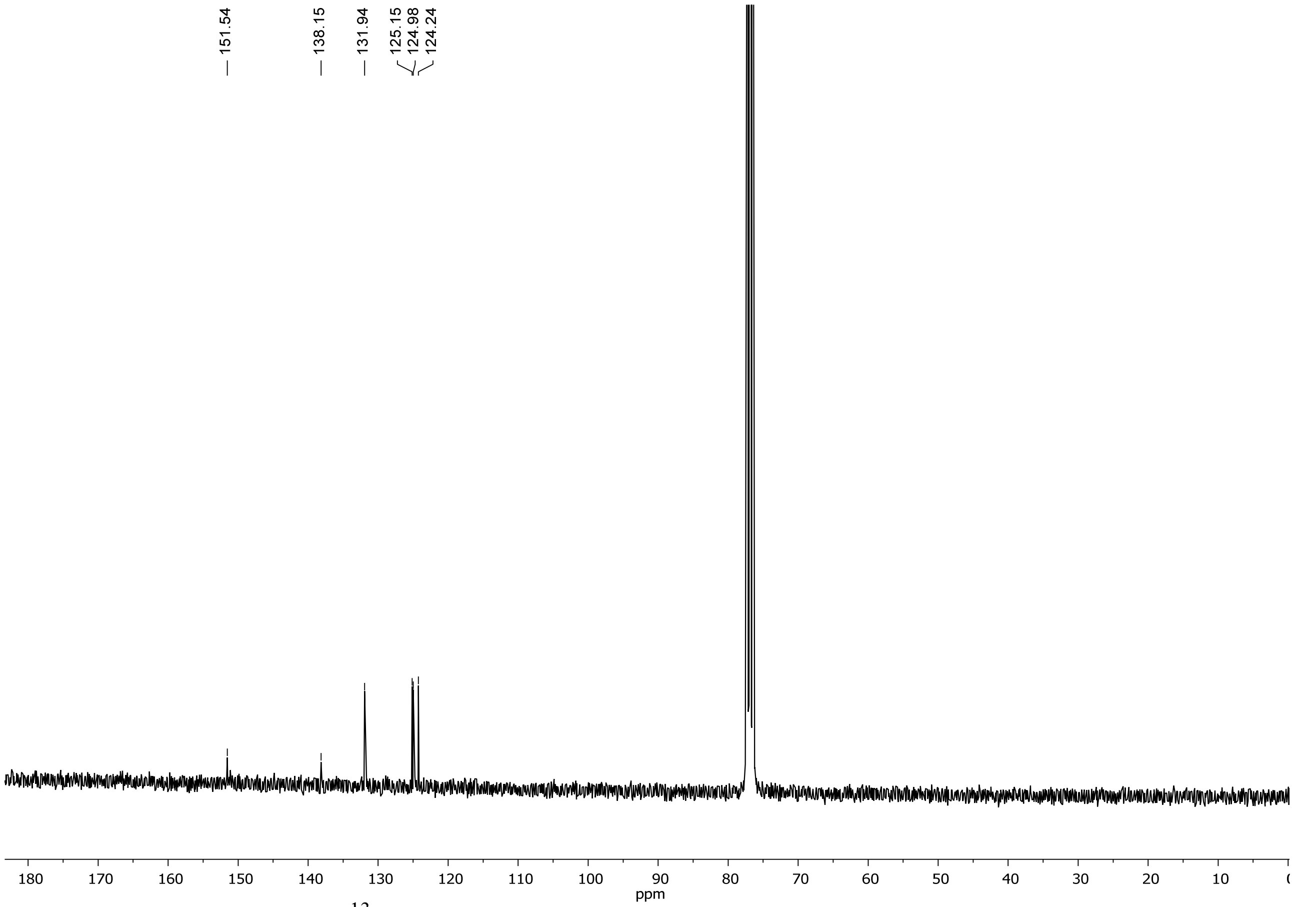

Figure part S.1.d. ^{13}C NMR spectra of compound **16** in $CDCl_3$ (75 MHz).

S.2 Interaction with CT DNA

The interaction of the compounds with CT DNA was investigated by UV-vis spectroscopy, viscosity measurements and fluorescence emission spectroscopy studies.

S.2.1 Binding study with CT DNA by UV-vis spectroscopy

UV-vis spectroscopy was used for the evaluation of the interaction of the compounds with CT DNA, and specifically the possible binding modes of the compounds to CT DNA. Control experiments with DMSO were performed and no changes in the spectra of CT DNA were observed.

In order to determine the binding mode, the UV-vis spectra of the compounds were recorded for a constant concentration (5×10^{-5} - 10^{-4} M) at the corresponding λ_{max} with increasing concentrations of CT DNA for diverse *r* (*r* = [complex]/[DNA]) values. Effective use of the changes in the absorbance of the UV-vis spectra was made and the DNA-binding constants of the compounds (K_b, M^{-1}) were calculated by the Wolfe-Shimer equation (eq. 1) [1] and the plots $[DNA]/(\varepsilon_A-\varepsilon_f)$ *versus* [DNA]:

$$\frac{[DNA]}{(\varepsilon_A-\varepsilon_f)}=\frac{[DNA]}{(\varepsilon_b-\varepsilon_f)}+\frac{1}{K_b(\varepsilon_b-\varepsilon_f)} \quad \text{(eq 1)}$$

where [DNA] = the concentration of DNA in base pairs, ε_f = the extinction coefficient for the free compound at the corresponding λ_{max}, $\varepsilon_A = A_{obsd}$/[compound] and ε_b = the extinction coefficient for the compound in the fully bound form. K_b is given by the ratio of slope to the y intercept in plots $[DNA]/(\varepsilon_A-\varepsilon_f)$ *versus* [DNA].

S.2.2 CT DNA-binding studies by viscosity measurements

The viscosity of DNA (0.1 mM) in buffer solution was measured in the absence and presence of increasing amounts of the compounds. The experiments were executed at room temperature and the measurements are devised in a plot $(\eta/\eta_0)^{1/3}$ *versus* r (*r* = [complex]/[DNA]), where η = the viscosity of DNA in the presence of the compound, and η_0 = the viscosity of DNA in buffer solution.

S.2.3 EB-displacement studies

In order to determine and confirm the DNA-binding mode of the compounds, a competitive study with EB as an intercalating marker is performed by fluorescence emission spectroscopy. Therefore, the EB-displacing ability of the compounds from its EB-DNA conjugate was examined.

The DNA-EB adduct was prepared by addition of 20 μM EB and 26 μM CT DNA in buffer solution (150 mM NaCl and 15 mM trisodium citrate at pH 7.0). The potential intercalation of the compounds between the DNA-bases was studied by the addition of a certain amount of the compound solution into the EB-DNA adduct solution. The influence of the compounds on the EB-DNA solution was monitored through the changes of the fluorescence emission spectra at excitation wavelength (λ_{ex}) at 540 nm [2]. The tested compounds do not show any significant fluorescence at room temperature in solution or in the presence of DNA, under the same experimental conditions (λ_{ex} = 540 nm). Bearing that in mind, the observed quenching of the EB-DNA solution is evidently associated to the displacement of EB from its EB-DNA adduct.

The quenching efficiency (K_{SV}) for each compound was assessed according to the Stern-Volmer equation (eq. 2) [2]:

$$\frac{Io}{I} = 1 + k_q \tau_0 [Q] = 1 + K_{SV}[Q] \quad \text{(eq. 2)}$$

where I_0 and I = the fluorescence emission intensities of EB-DNA in the absence and presence of the quencher, respectively, [Q] = the concentration of the quencher (i.e. compounds). K_{SV} is obtained from the Stern-Volmer plots by the slope of the diagram I_0/I *versus* [Q]. Taking τ_0 = 23 ns as the fluorescence lifetime of the EB-DNA adduct [3], the EB-DNA quenching constants (k_q, in $M^{-1}s^{-1}$) of the compounds can be determined according to equation 3:

$$K_{SV} = k_q \tau_o \quad \text{(eq. 3)}$$

S.2.4 References

1. Wolfe, A.; Shimer, G.H.; Meehan, T. Polycyclic Aromatic Hydrocarbons Physically Intercalate into Duplex Regions of Denatured DNA. *Biochemistry* **1987**, *26*, 6392–6396, doi:10.1021/bi00394a013.
2. Lakowicz, J.. R. *Principles of Fluorescence Spectroscopy*; Springer New York, NY, 2006; ISBN 978-0-387-31278-1.
3. Heller, D.P.; Greenstock, C.L. Fluorescence lifetime analysis of DNA intercalated ethidium bromide and quenching by free dye. *Biophys. Chem.* **1994**, *50*, 305–312, doi:10.1016/0301-4622(93)E0101-A.

S.3 Plot of $\frac{[DNA]}{(\varepsilon_A - \varepsilon_f)}$ *versus [DNA] of compounds 1-17*

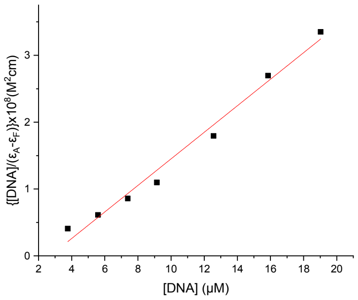

1

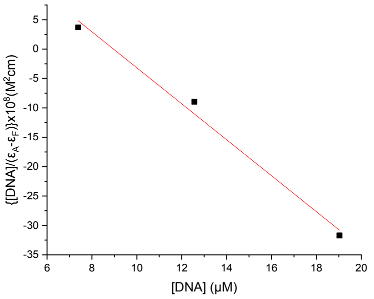

2

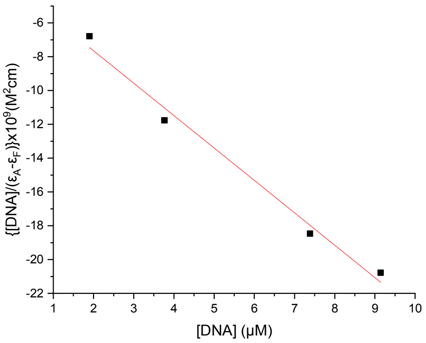

3

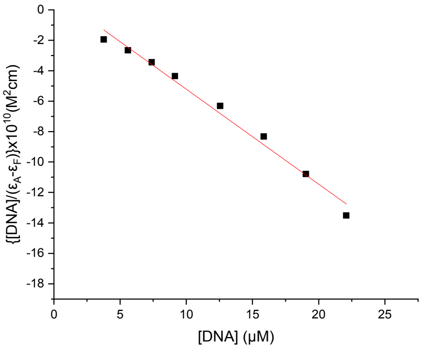

4

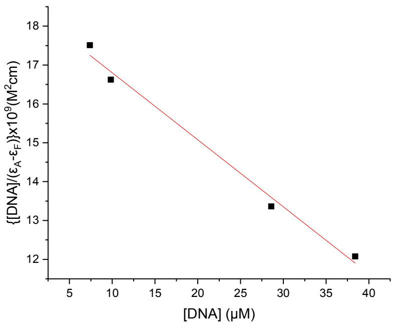

5

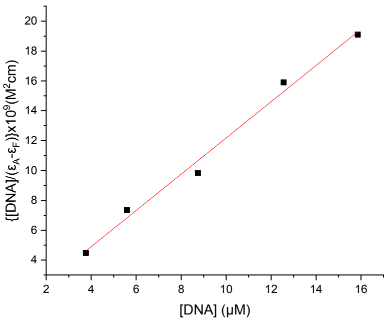

6

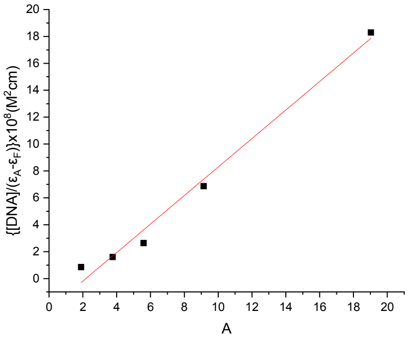

7

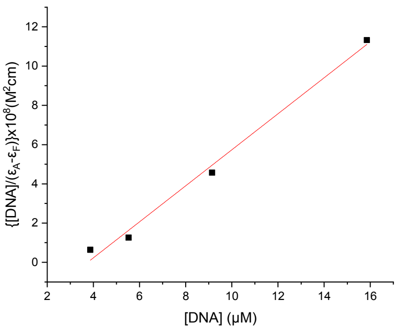

8

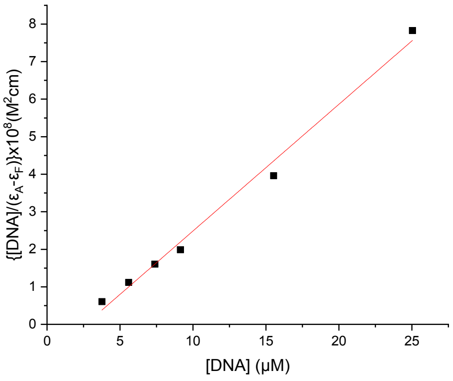

9

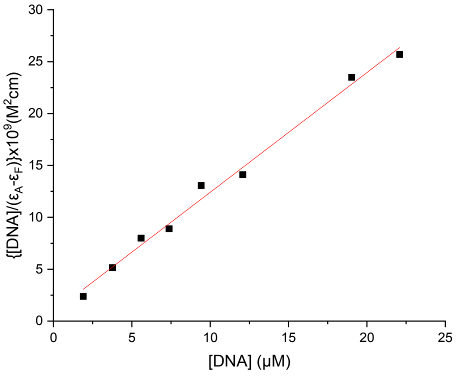

10

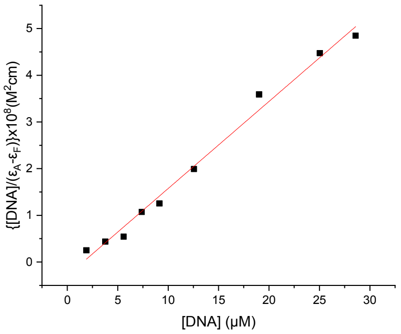

11

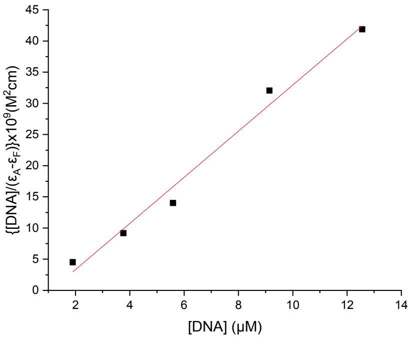

12

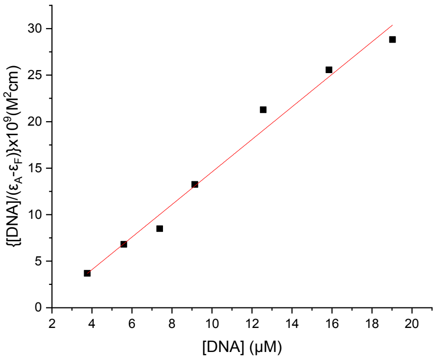

13

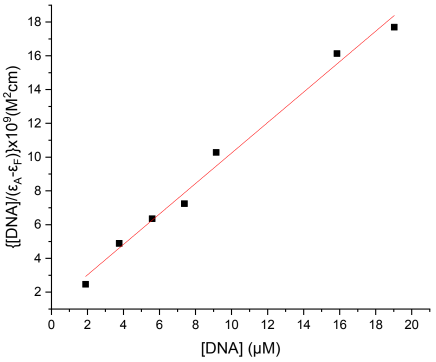

14

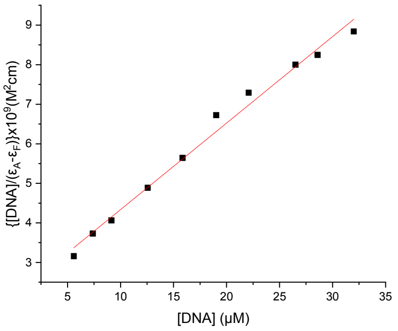

15

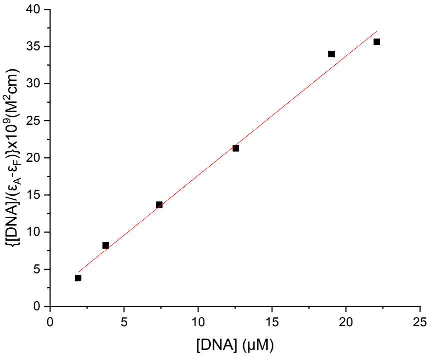

16

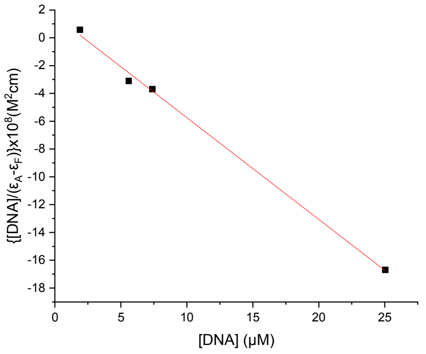

17

Figure part S.3. Plot of $\frac{[\mathrm{DNA}]}{(\varepsilon_A - \varepsilon_f)}$ *versus* [DNA] for compounds **1-17.**

S.4 Stern–Volmer quenching plot of EB–DNA fluorescence of compounds 1-17.

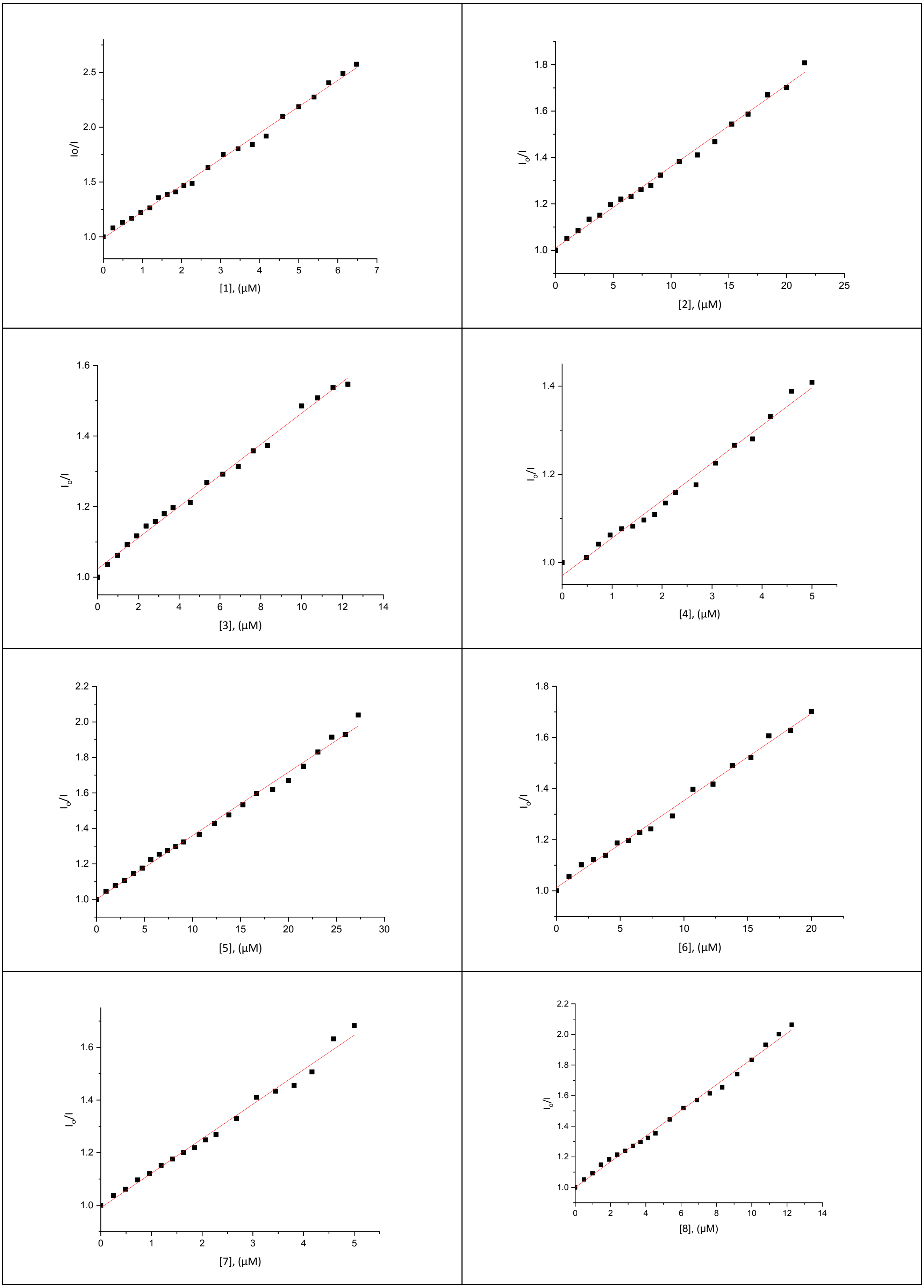

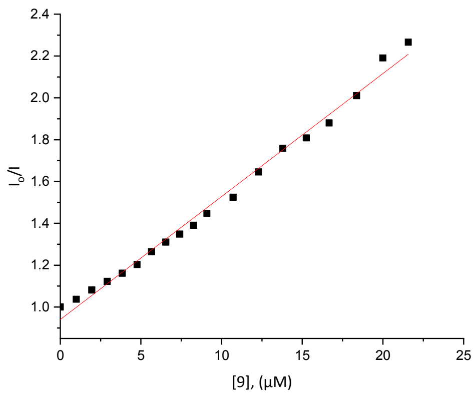
I_o/I
[9], (μM)

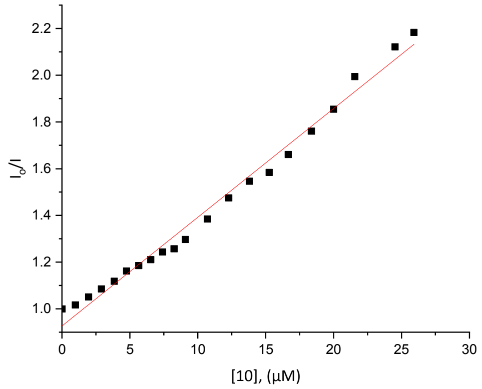
I_o/I
[10], (μM)

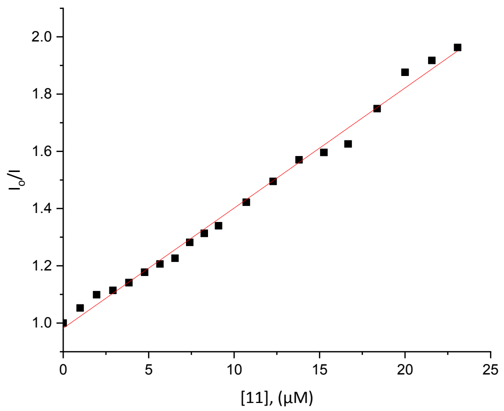
I_o/I
[11], (μM)

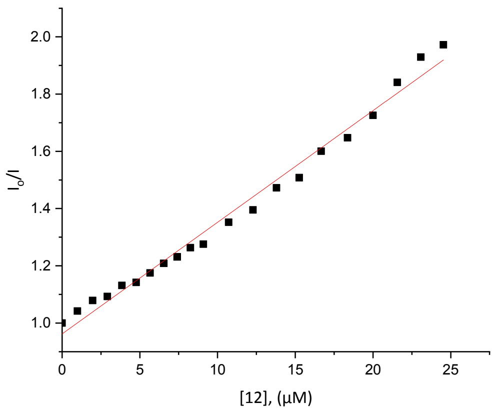
I_o/I
[12], (μM)

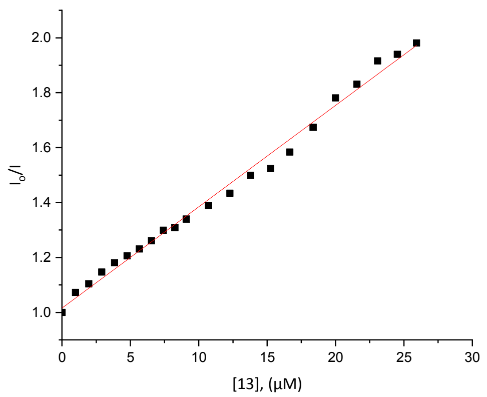
I_o/I
[13], (μM)

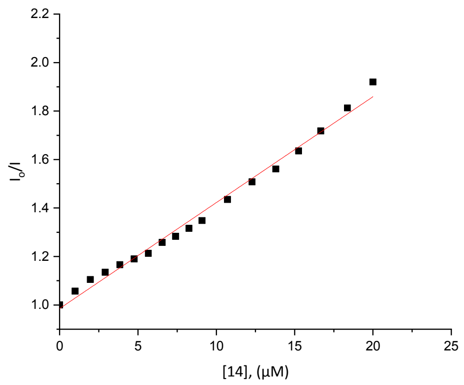
I_o/I
[14], (μM)

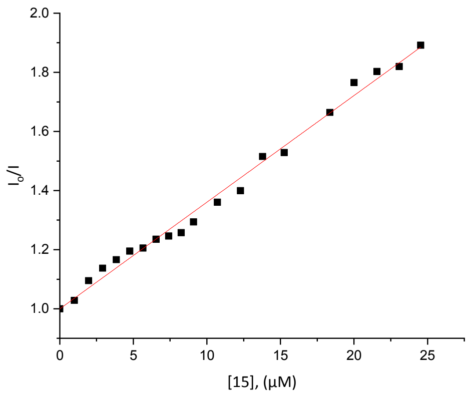
I_o/I
[15], (μM)

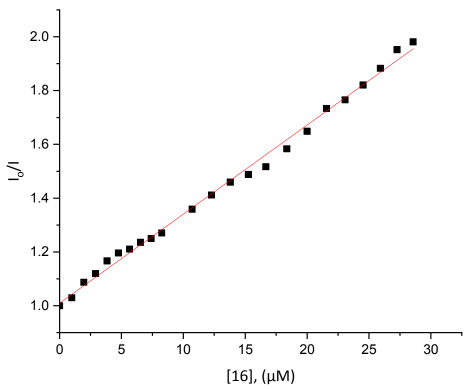
I_o/I
[16], (μM)

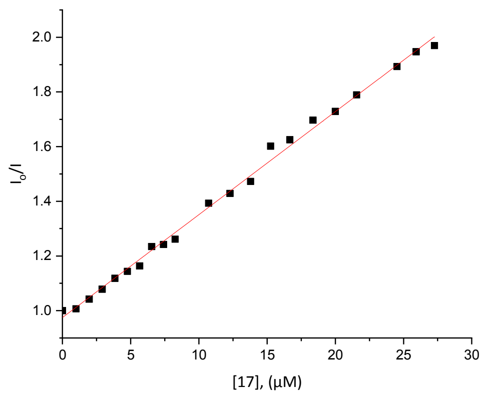

Figure part S.4. Stern–Volmer quenching plot of EB–DNA fluorescence for compounds **1-17.**

*S.5 Copies of NMR spectra of **1**, **5** and **8** in various solvents and time intervals*

***S.5.1** Compound **1** (DMSO-d_6, DMSO-d_6 & D_2O, CD_3OD)*

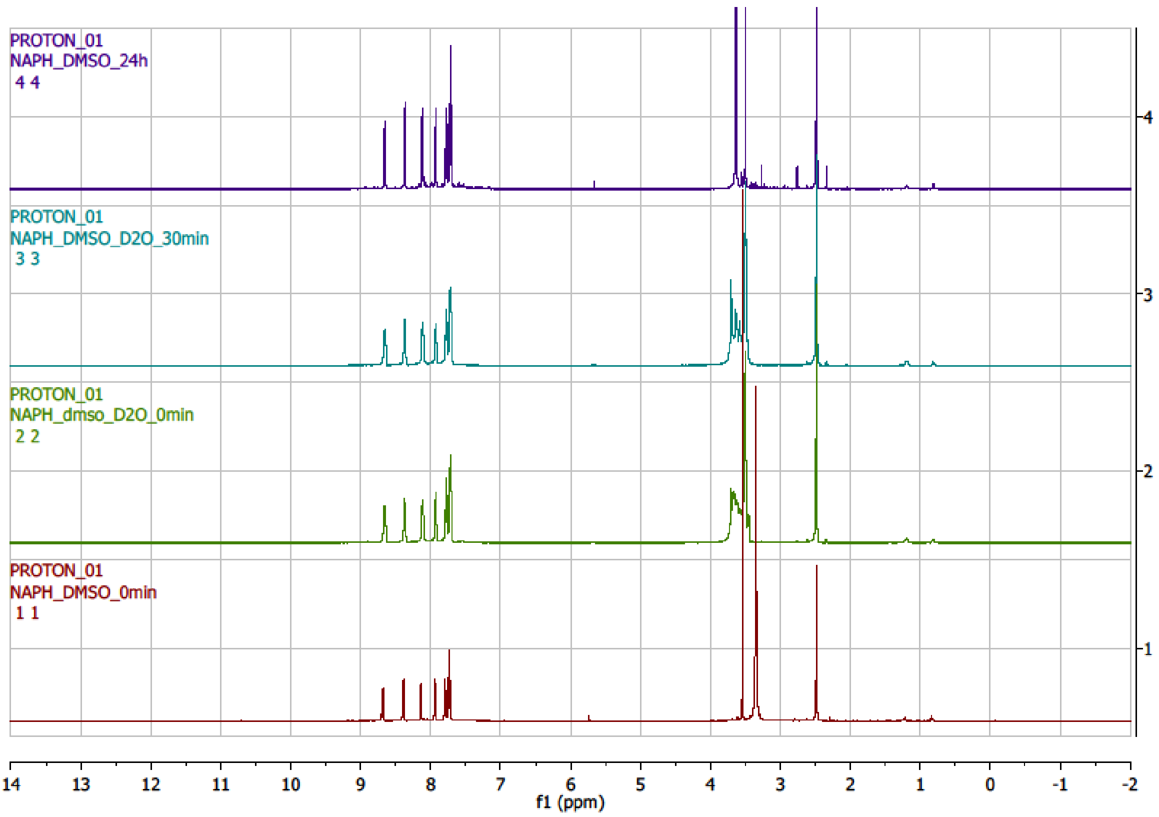

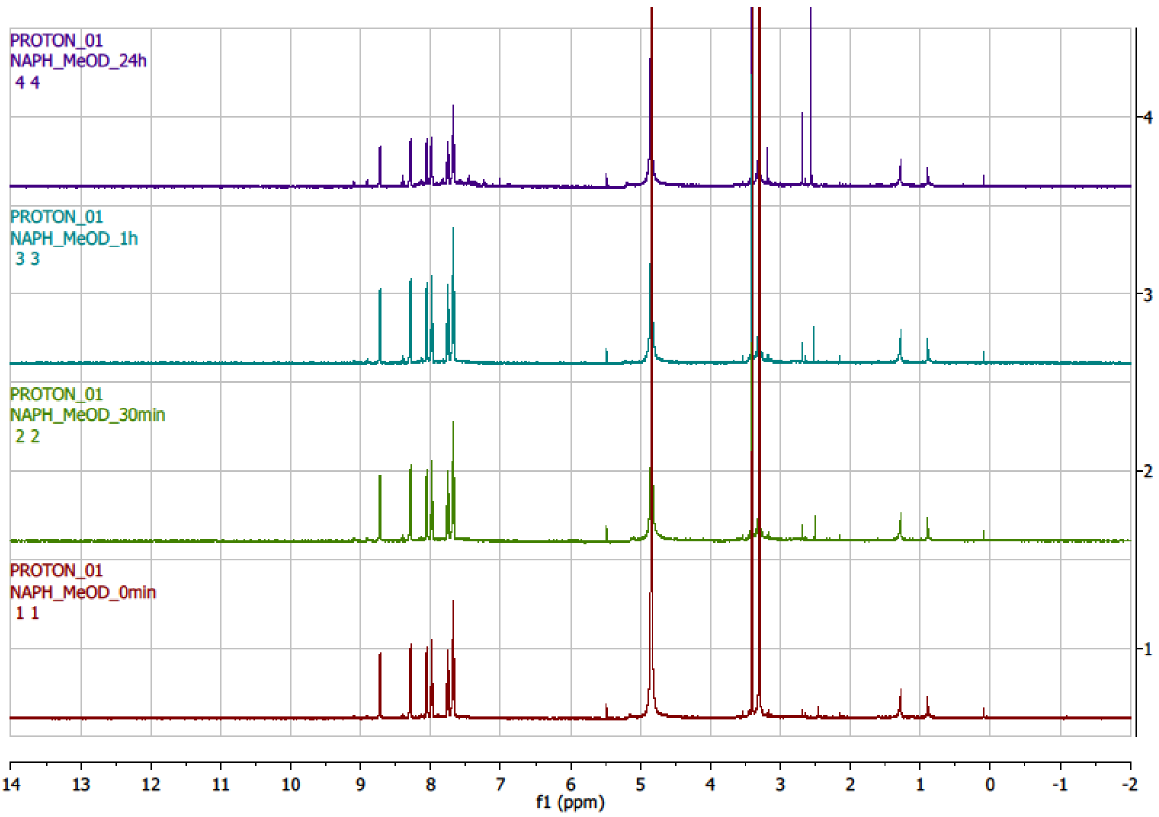

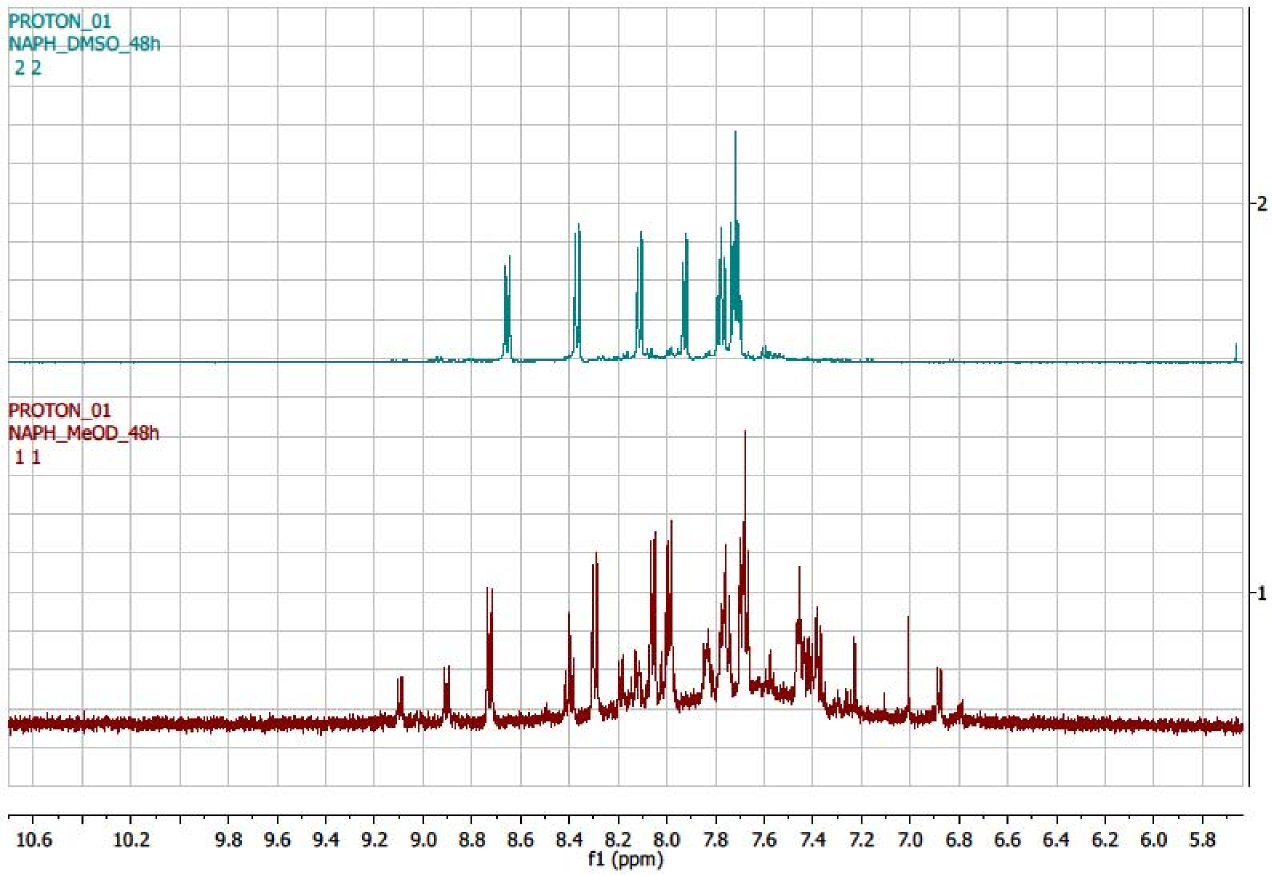
PROTON_01
NAPH_DMSO_48h
2 2
PROTON_01
NAPH_MeOD_48h
1 1
2
1
10.6
10.2
9.8
9.6
9.4
9.2
9.0
8.8
8.6
8.4
8.2
8.0
7.8
7.6
7.4
7.2
7.0
6.8
6.6
6.4
6.2
6.0
5.8
f1 (ppm)

***S.5.2** Compound **5** (DMSO-d_6, DMSO-d_6 & D_2O, CD_3OD)*

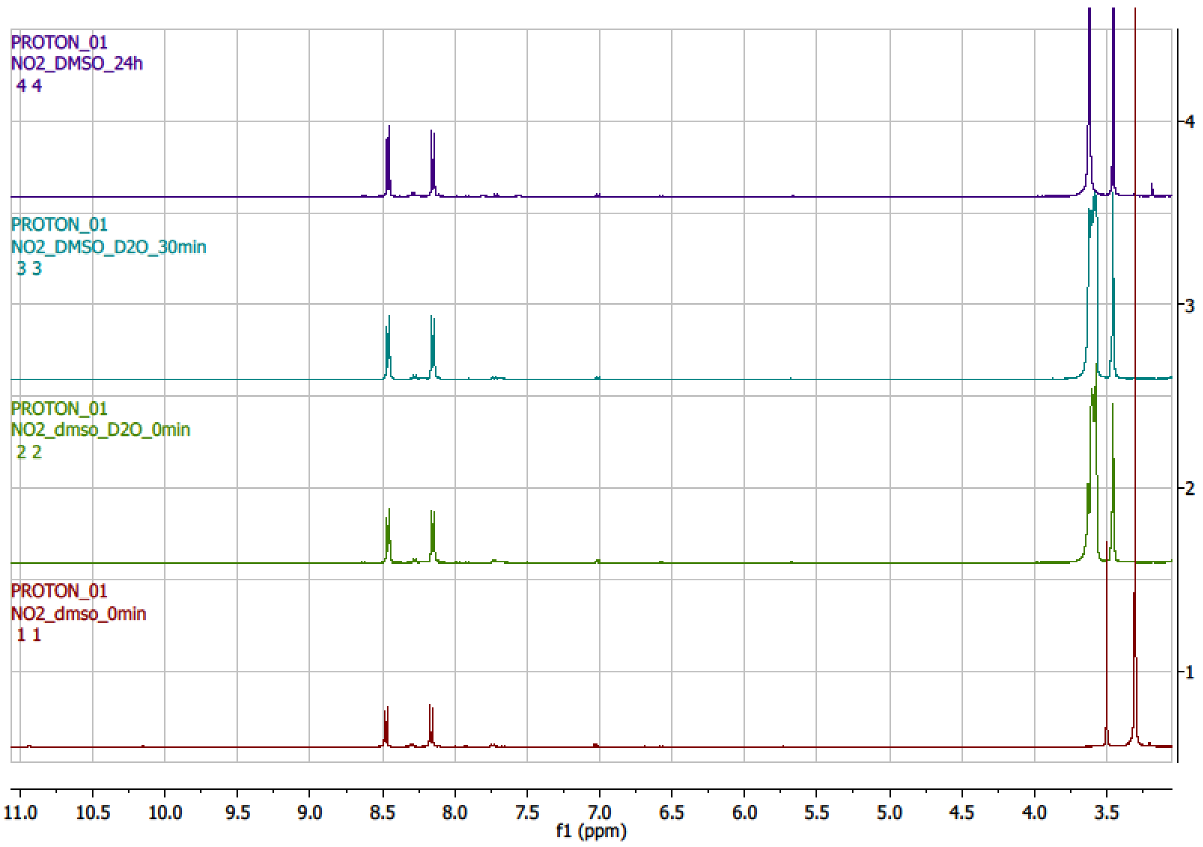

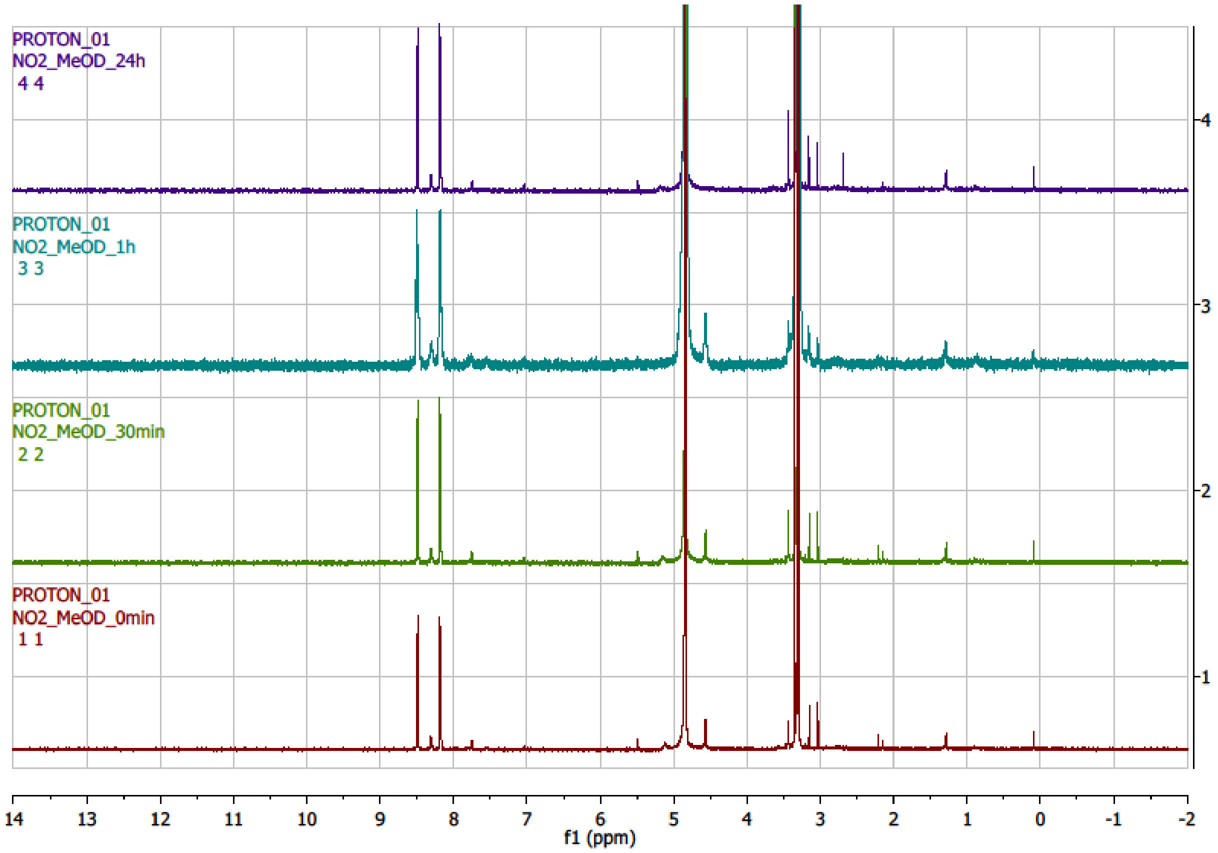

S.5.3 Compound 8 (DMSO-d6, DMSO-d6 & D2O, CD3OD)

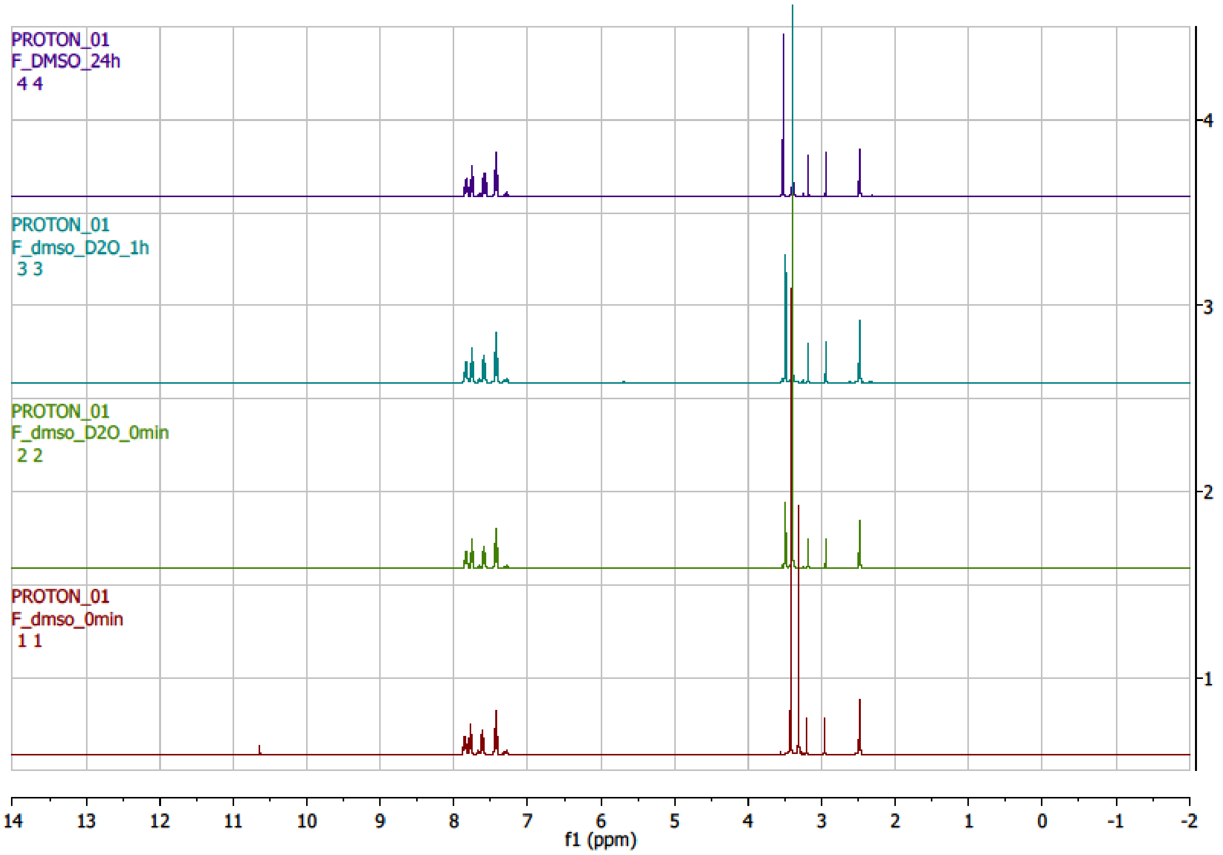

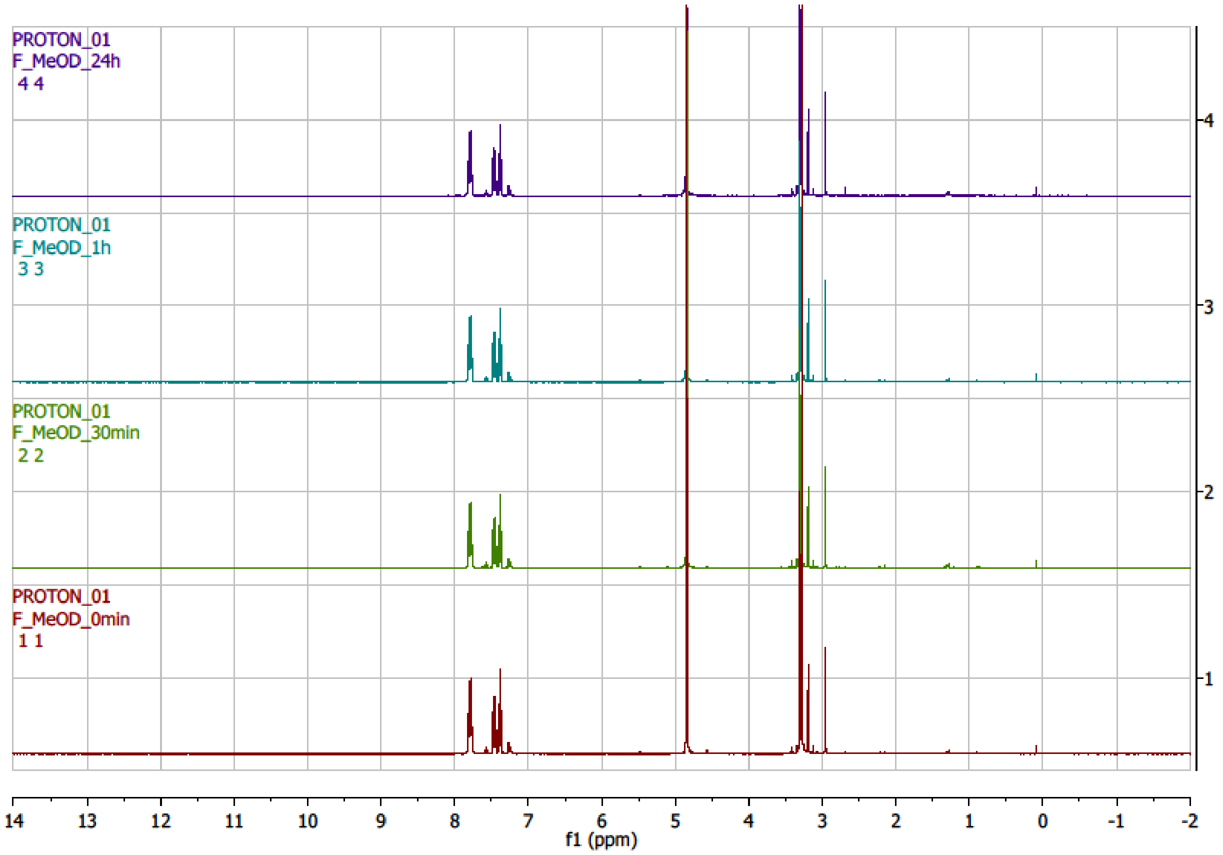

S.6 Gel electrophoresis pictures

S.6.1 Agarose gel electrophoresis of compounds ***1-17*** *in dark*

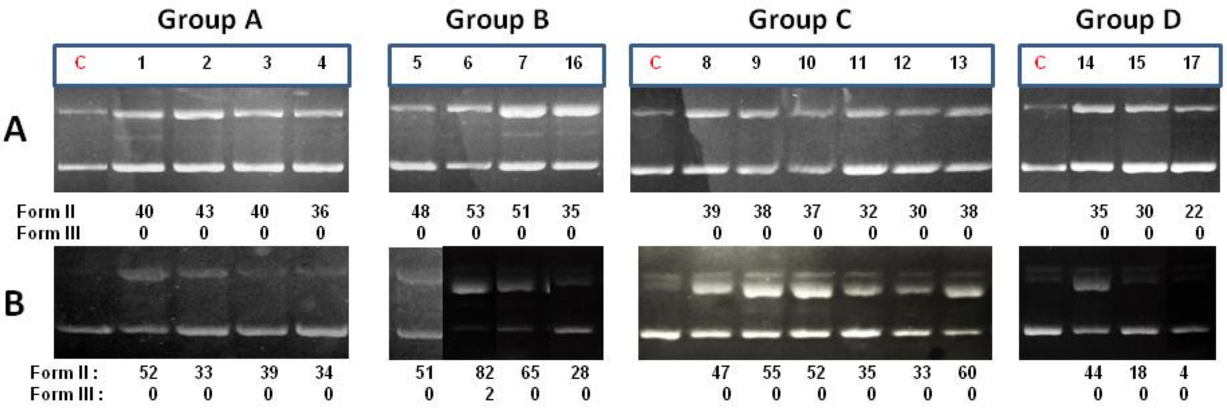

Figure, part S.6.1. Agarose gel electrophoresis images. Compounds **1-17**, in dark **(A):** 30 min, 100 µM; **(B):** 120 min, 100 µM; **Blue square:** C = Control (pDNA without the compound); Numbers in blue frames = Numbers of the compounds in text; **Numbers below photos:** Form II = % ss DNA photo-cleavage (nicked), Form III = % ds DNA photo-cleavage (linear).

S.6.2 Agarose gel electrophoresis of compounds ***1-17*** *under UVA and visible light irradiation*

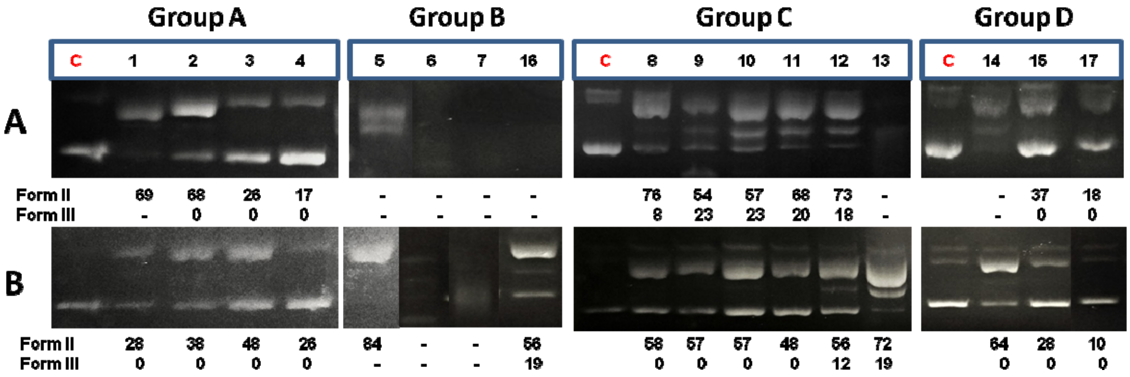

Figure, part S.6.2. Agarose gel electrophoresis images. Compounds **1-17**, 120 min, 100 µM: **(A):** UV-A irradiation; **(B):** Visible light irradiation. **Blue square:** C = Control (pDNA irradiated without the compound); Numbers in blue frames = Numbers of the compounds in text; **Numbers below photos:** Form II = % ss DNA photo-cleavage (nicked), Form III = % ds DNA photo-cleavage (linear), - not measurable plasmid due to extensive damage.

S.6.3 Agarose gel electrophoresis of compound ***5*** *in various concentrations under UVA light irradiation*

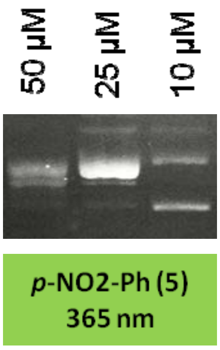

S.7 Copies of the UV-Vis spectra of compounds 1-17

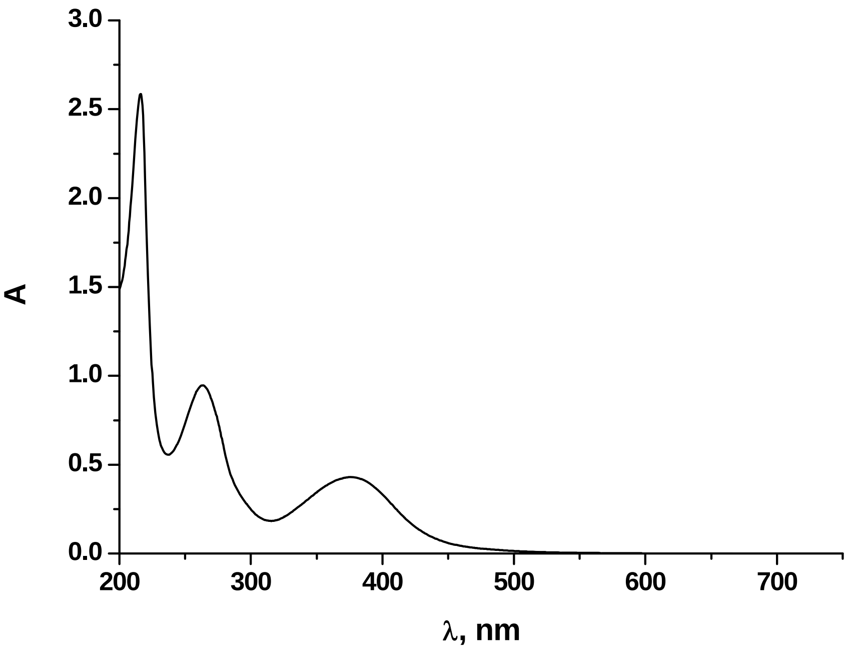

Figure part S.7.a. UV-Vis absorption spectra of **1** (5×10^{-5} M) in MeCN.

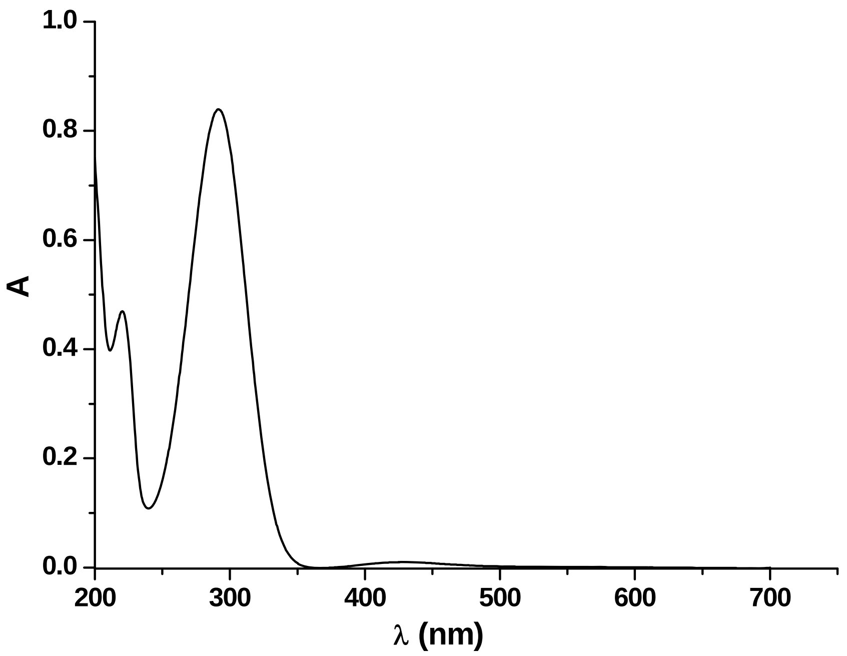

Figure part S.7.b1. UV-Vis absorption spectra of **2** (5×10^{-5} M) in MeCN.

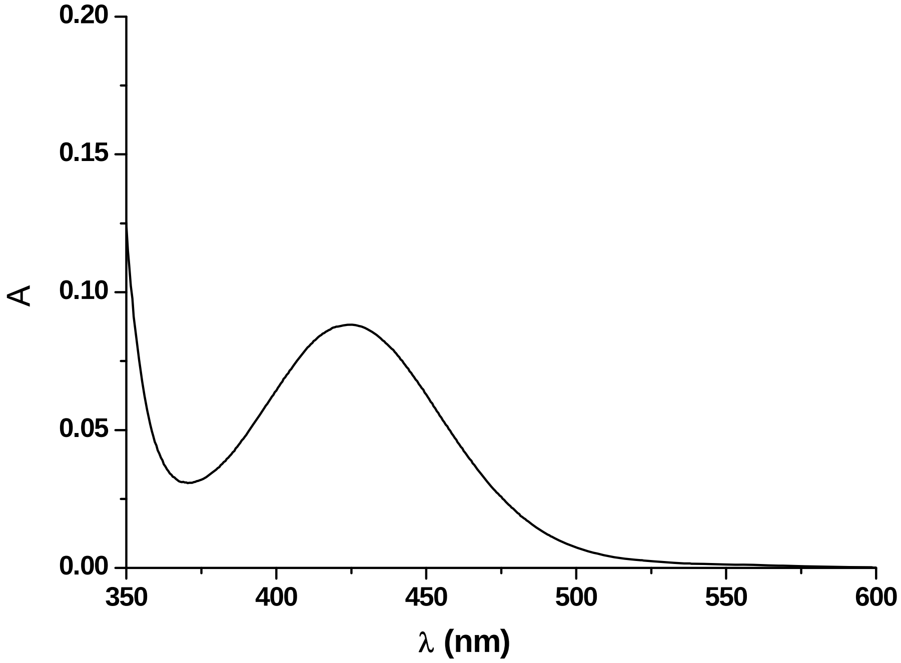

Figure part S.7.b2. UV-Vis absorption spectra of **2** (5×10^{-4} M) in MeCN.

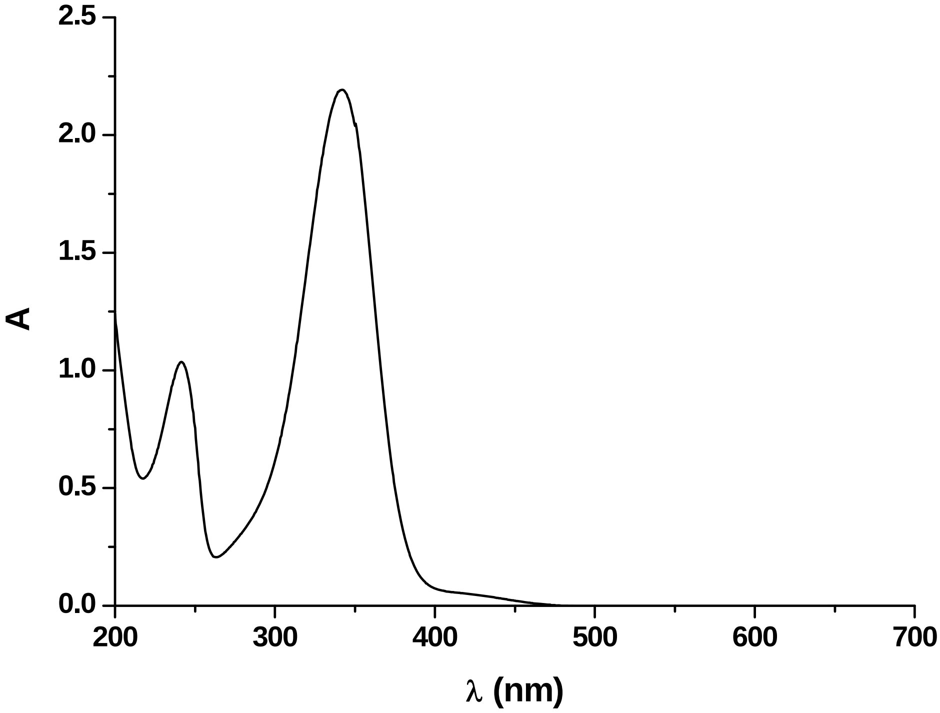

Figure part S.7.c. UV-Vis absorption spectra of **3** (5×10^{-5} M) in MeCN.

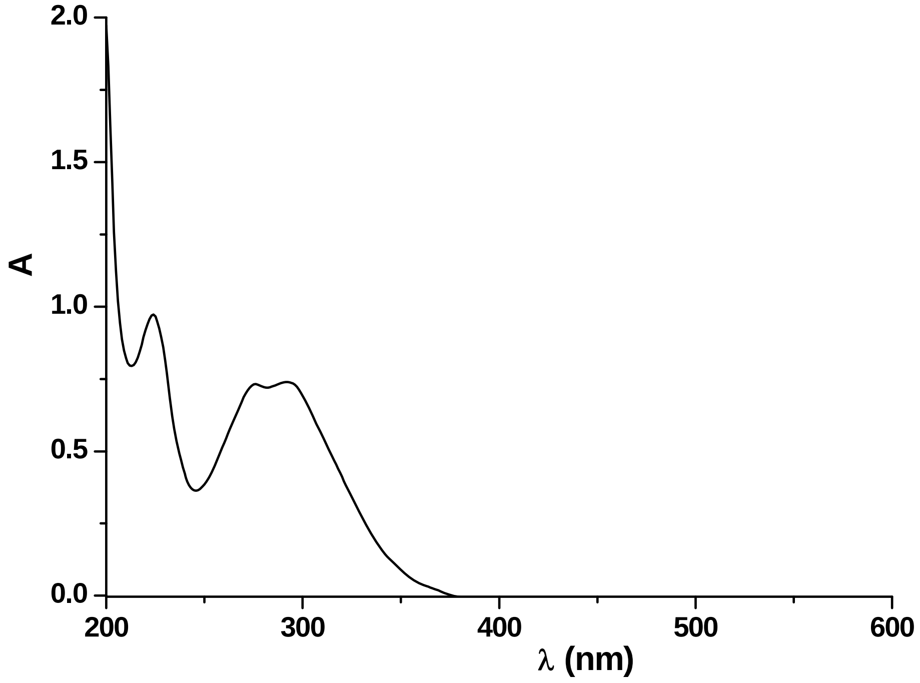

Figure part S.7.d1. UV-Vis absorption spectra of **4** (5×10^{-5} M) in MeCN.

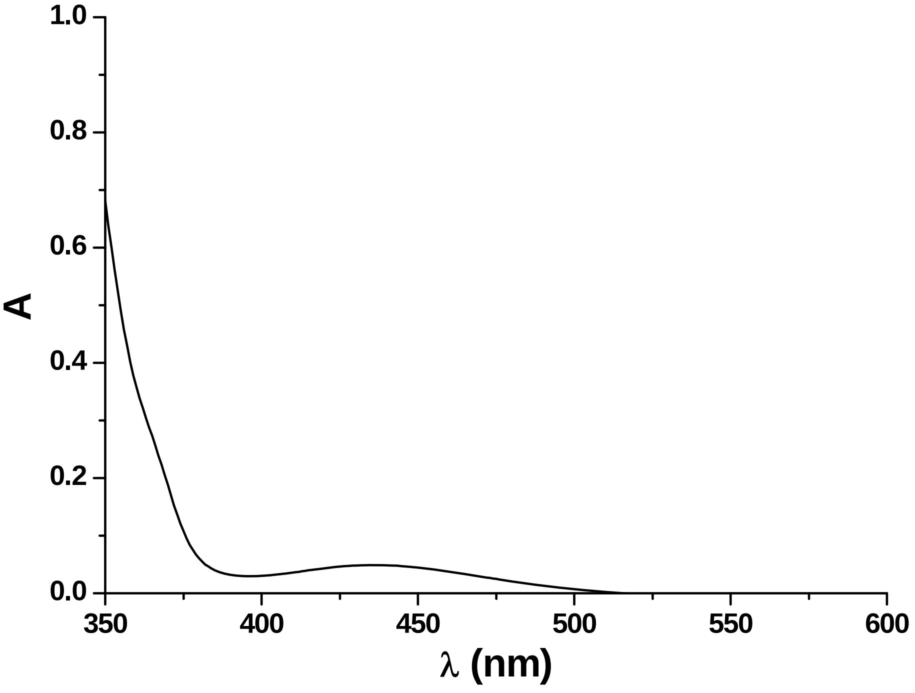

Figure part S.7.d2. UV-Vis absorption spectra of **4** (5×10^{-4} M) in MeCN.

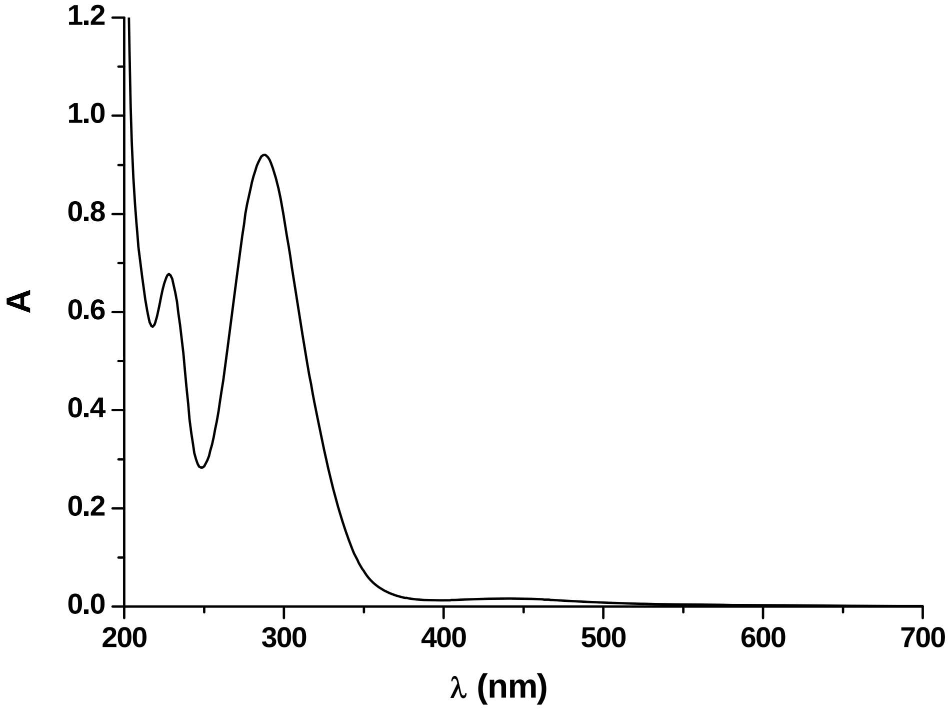

Figure part S.7.e. UV-Vis absorption spectra of **5** (5×10^{-5} M) in MeCN.

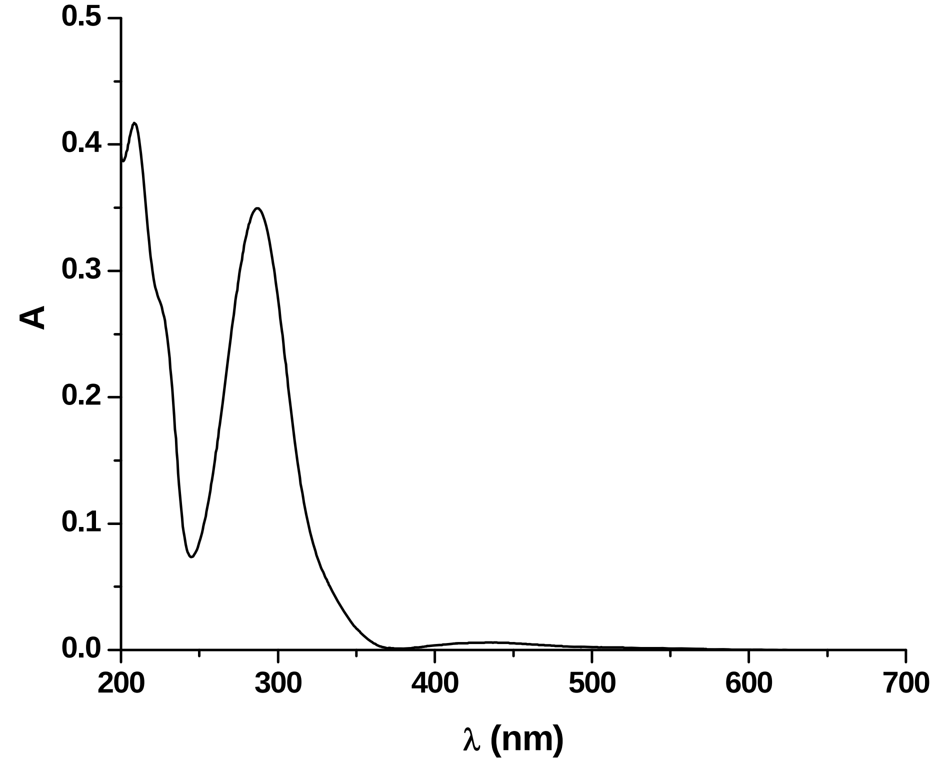

Figure part S.7.f. UV-Vis absorption spectra of **6** (5×10^{-5} M) in MeCN.

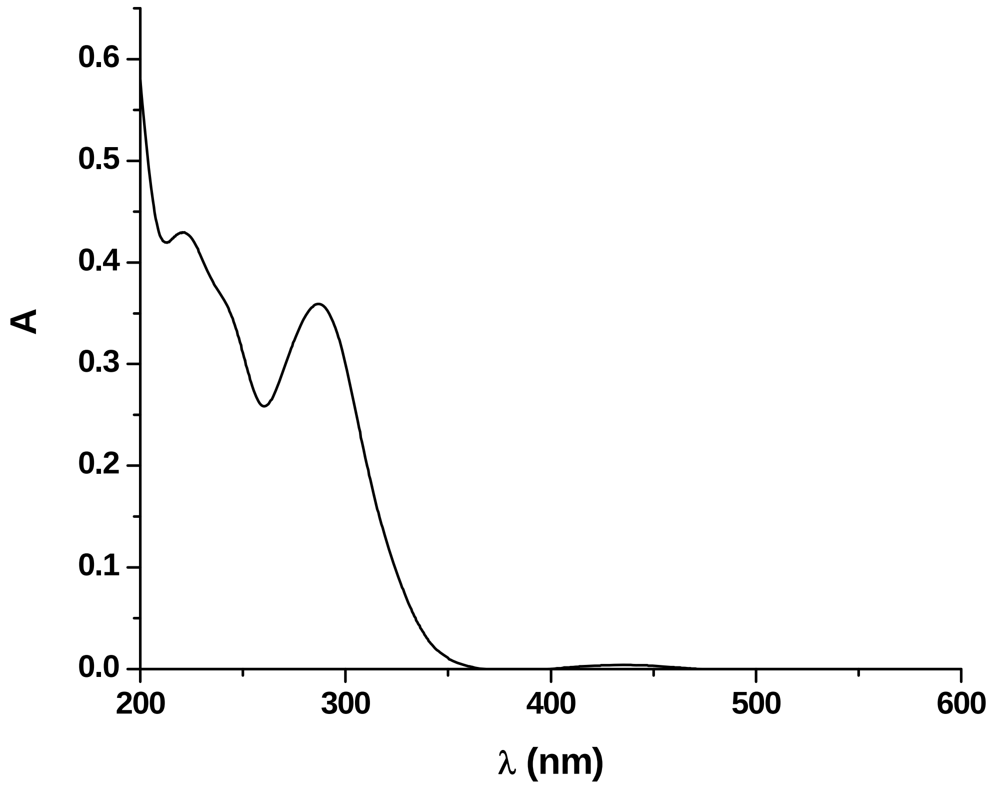

Figure part S.7.g1. UV-Vis absorption spectra of **7** (5×10^{-5} M) in MeCN.

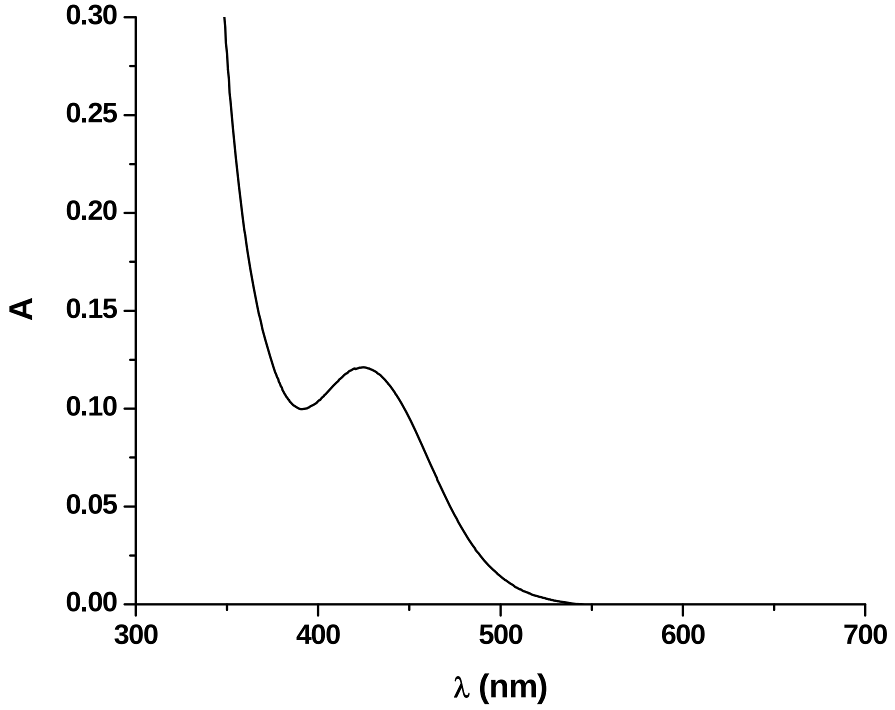

Figure part S.7.g2. UV-Vis absorption spectra of **7** (5×10^{-4} M) in MeCN.

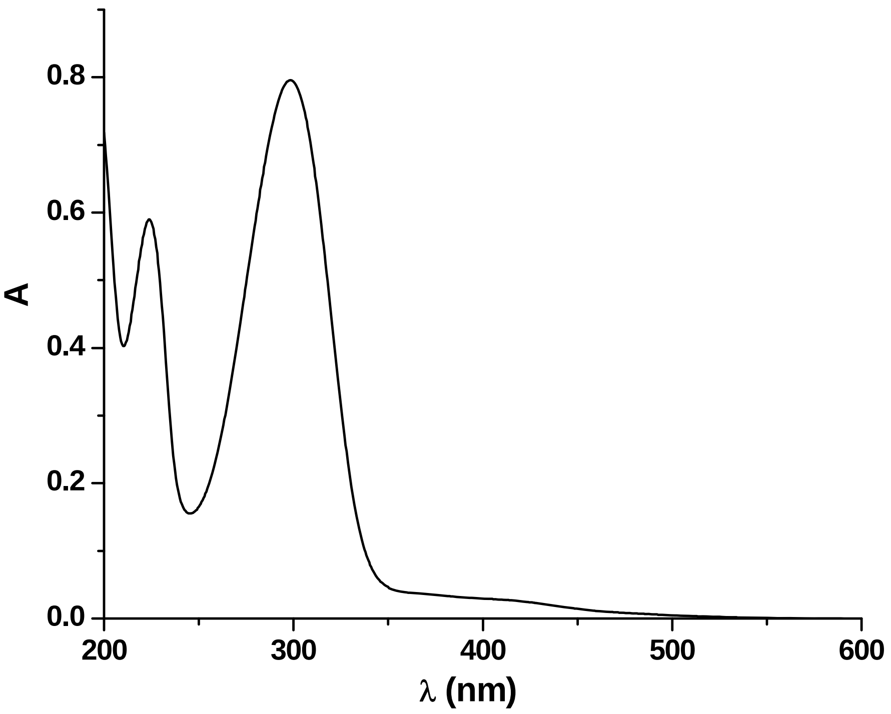

Figure part S.7.h.UV-Vis absorption spectra of **8** (5×10^{-5} M) in MeCN.

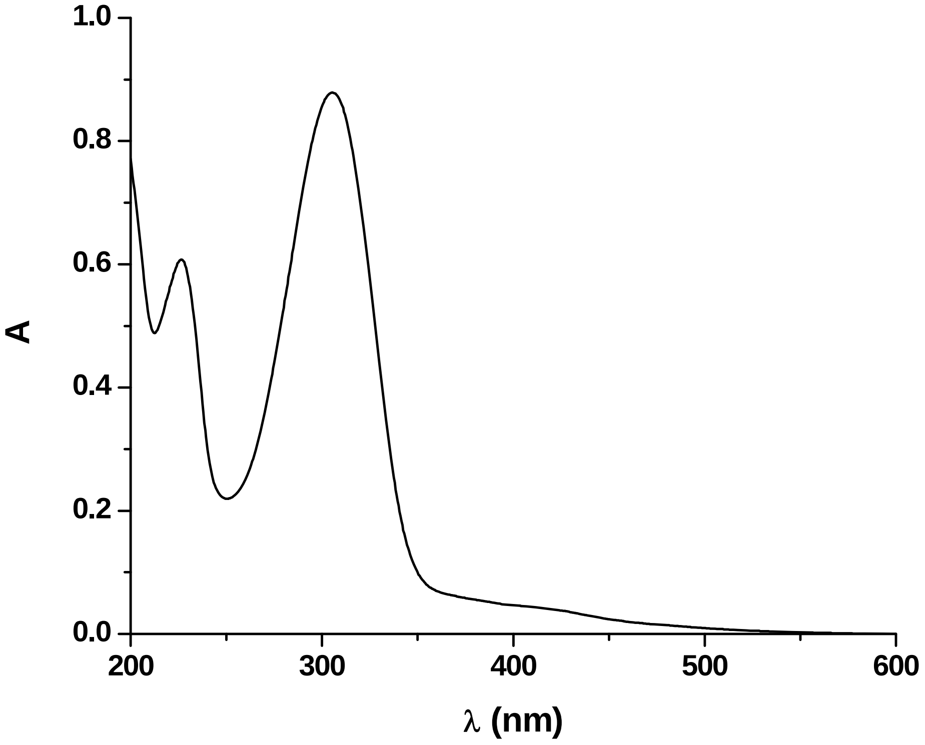

Figure part S.7.i. UV-Vis absorption spectra of **9** (5×10^{-5} M) in MeCN.

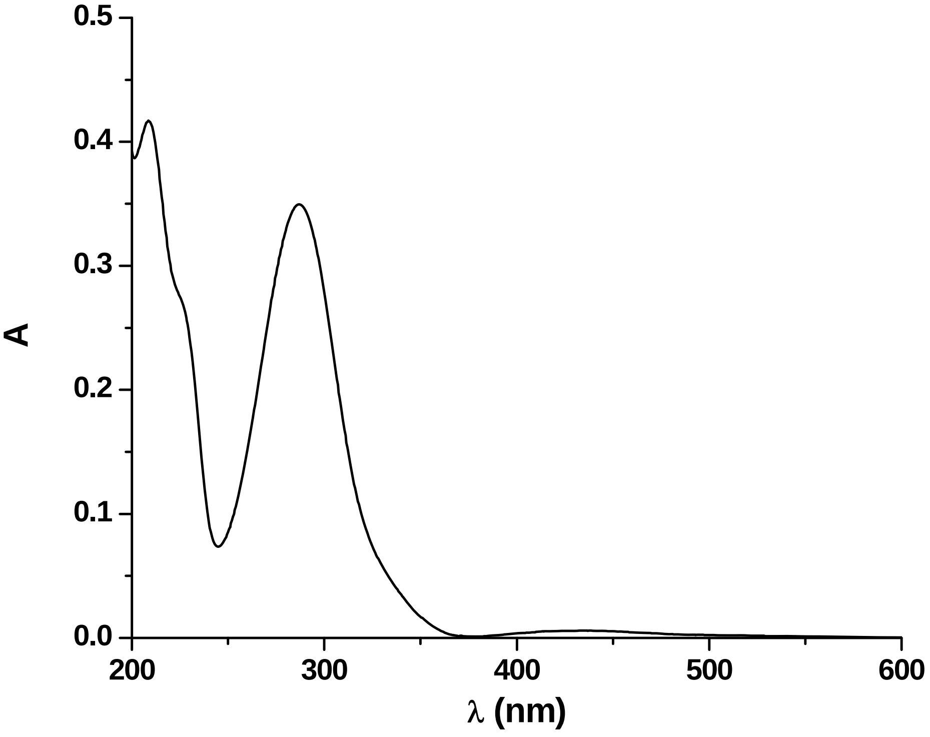

Figure part S.7.j. UV-Vis absorption spectra of **10** (5×10^{-5} M) in MeCN.

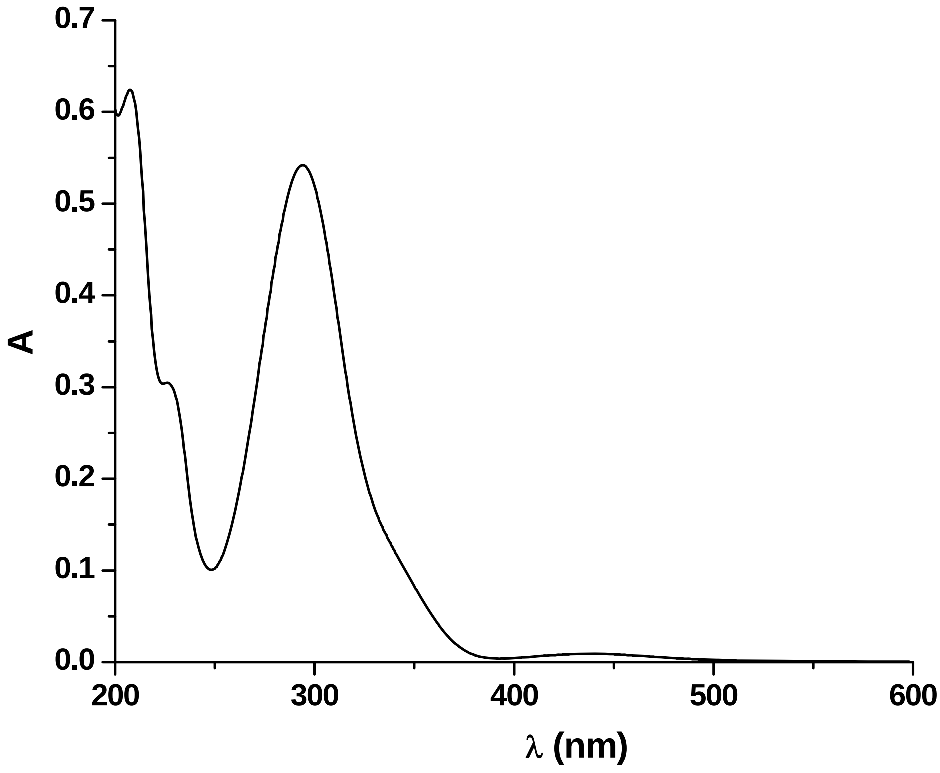

Figure part S.7.k. UV-Vis absorption spectra of **11** (5×10^{-5} M) in MeCN.

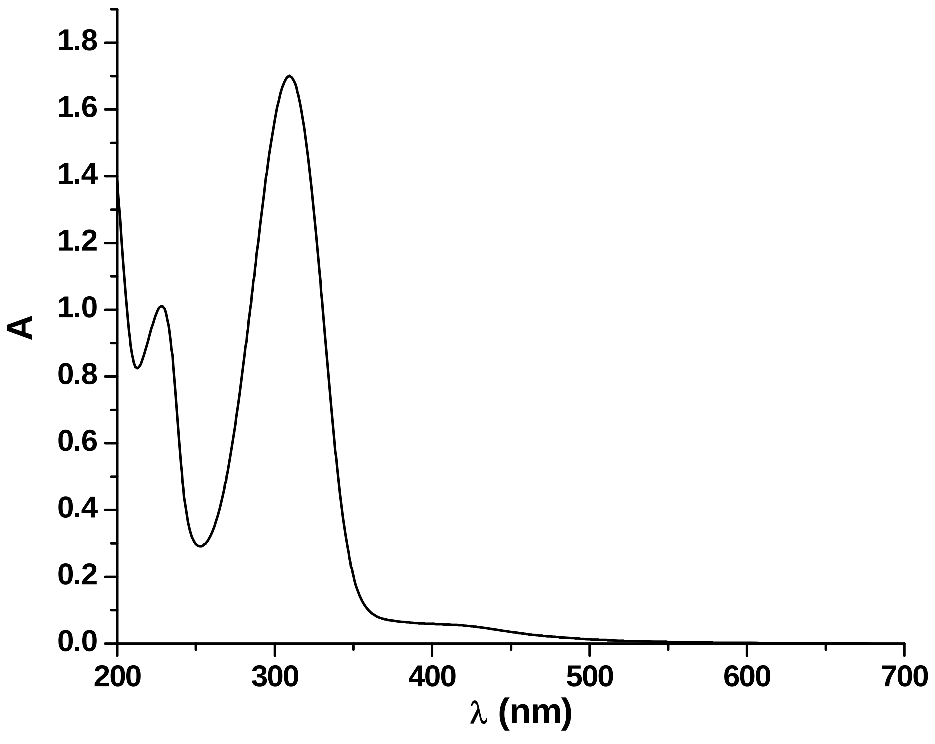

Figure part S.7.l. UV-Vis absorption spectra of **12** (5×10^{-5} M) in MeCN.

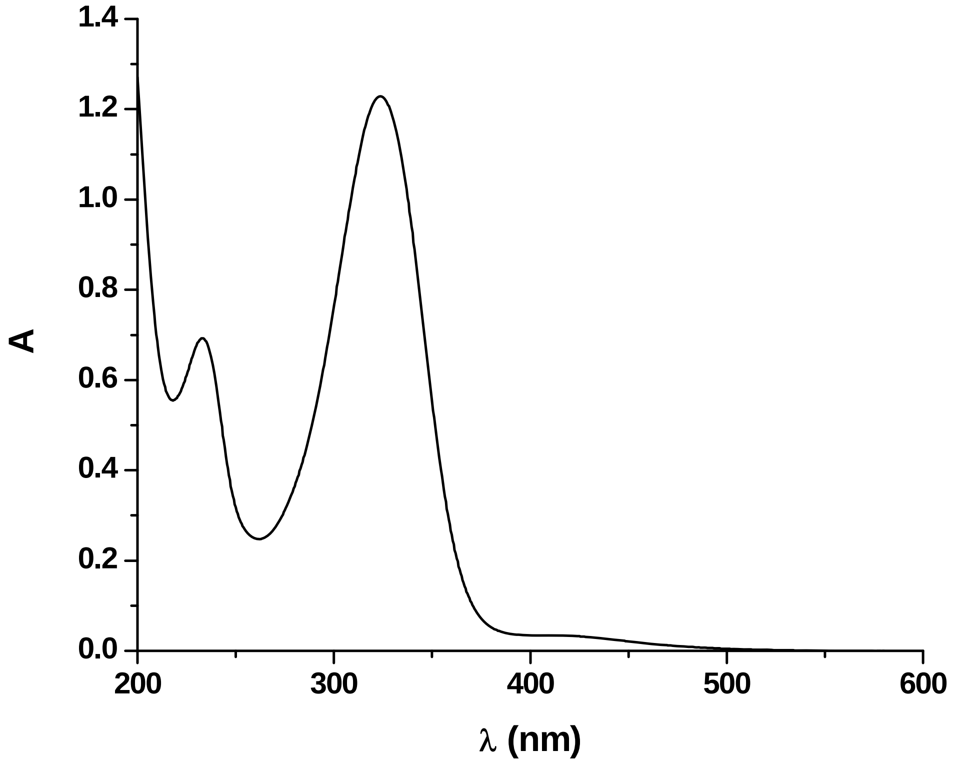

Figure part S.7.m. UV-Vis absorption spectra of **13** (5×10^{-5} M) in MeCN.

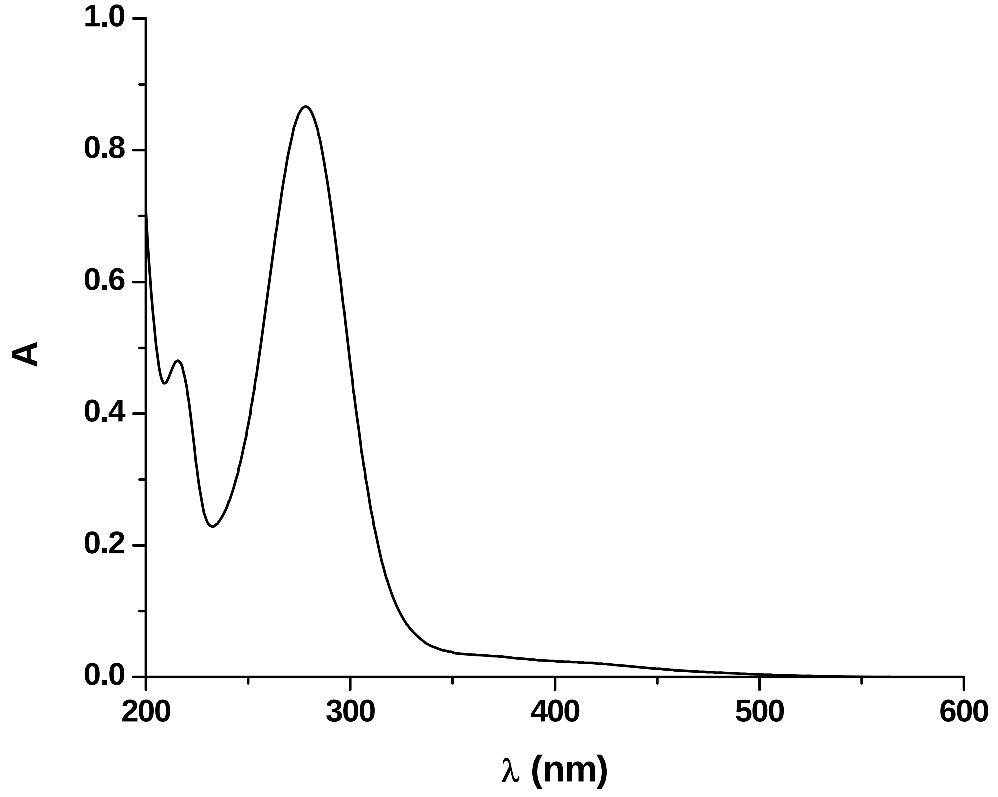

Figure part S.7.n. UV-Vis absorption spectra of **14** (5×10^{-5} M) in MeCN.

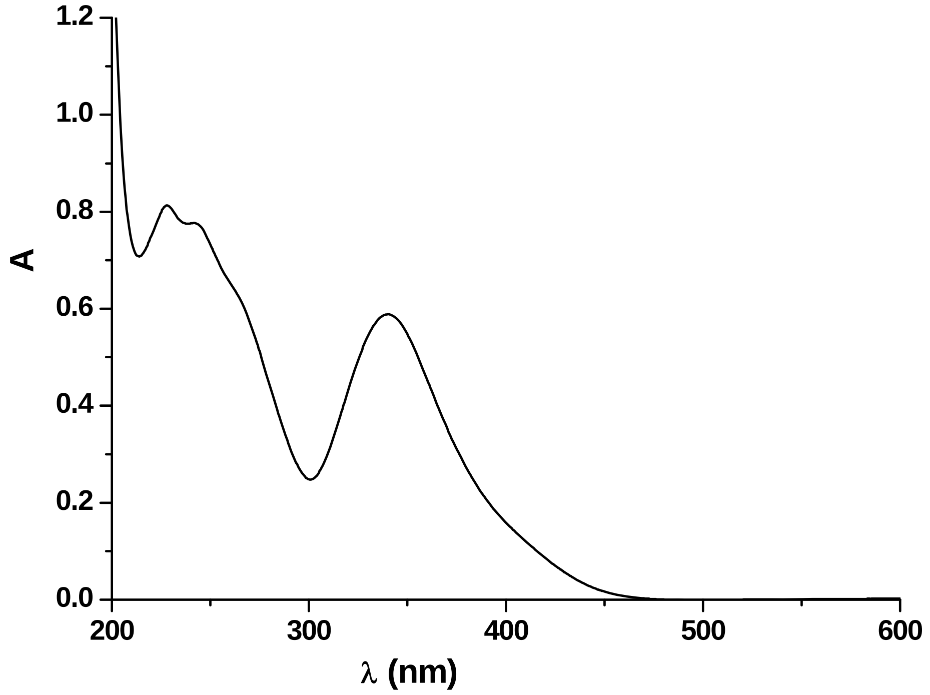

Figure part S.7.o. UV-Vis absorption spectra of **15** (5×10^{-5} M) in MeCN.

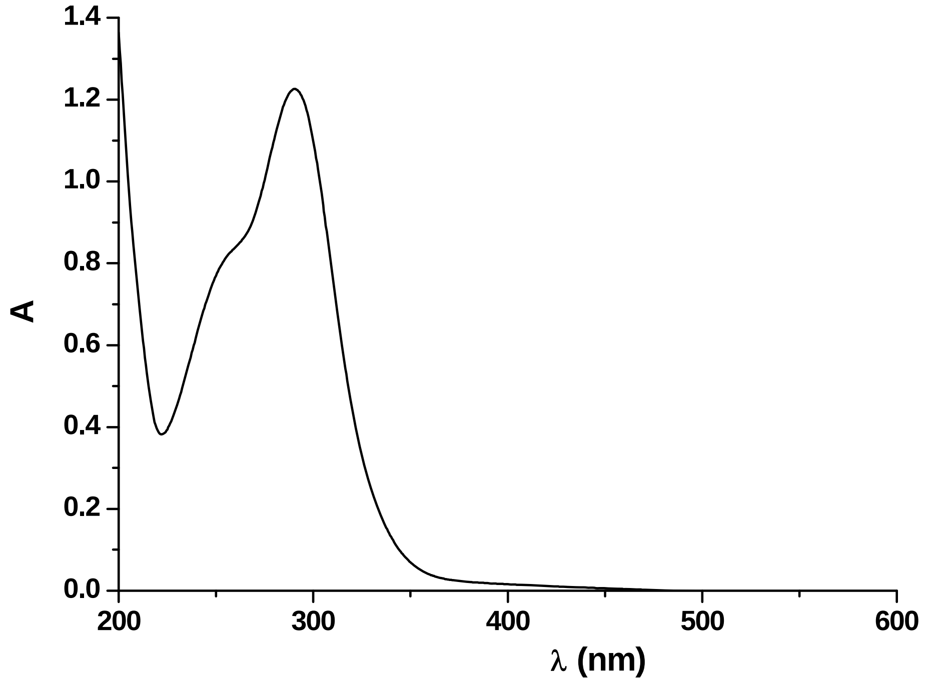

Figure part S.7.p.UV-Vis absorption spectra of **16** (5×10^{-5} M) in MeCN.

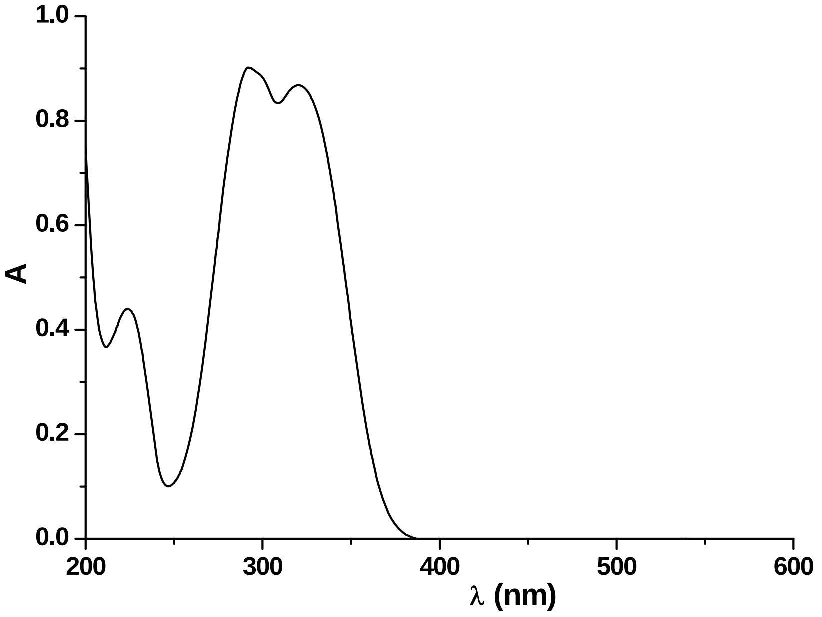

Figure part S.7.q1. UV-Vis absorption spectra of **17** (5×10^{-5} M) in MeCN.

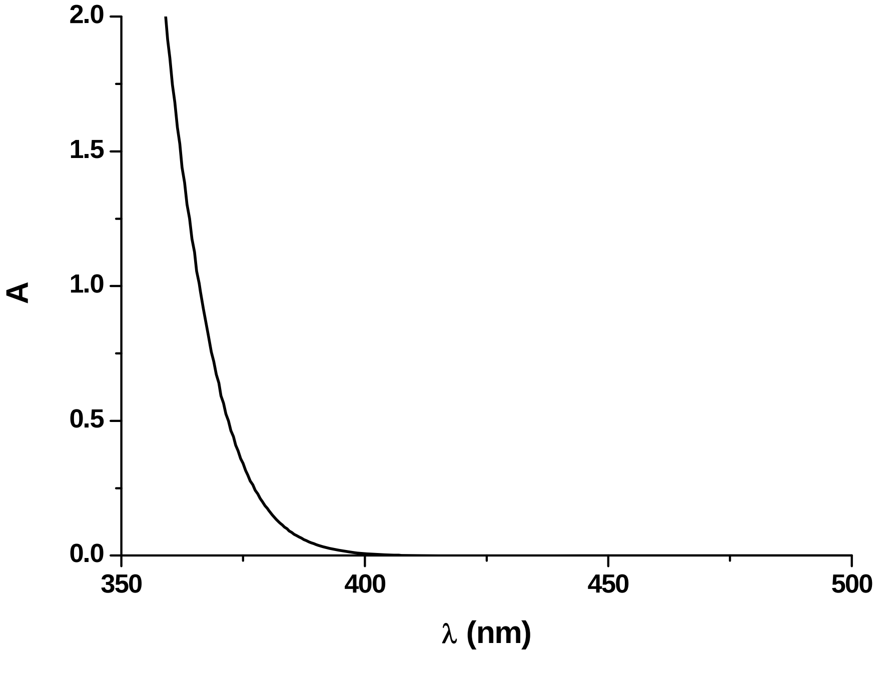

Figure part S.7.q2. UV-Vis absorption spectra of **17** (5×10^{-4} M) in MeCN.

S.8 Gel electrophoresis analysis (mechanistic studies) for compounds 1 and 5

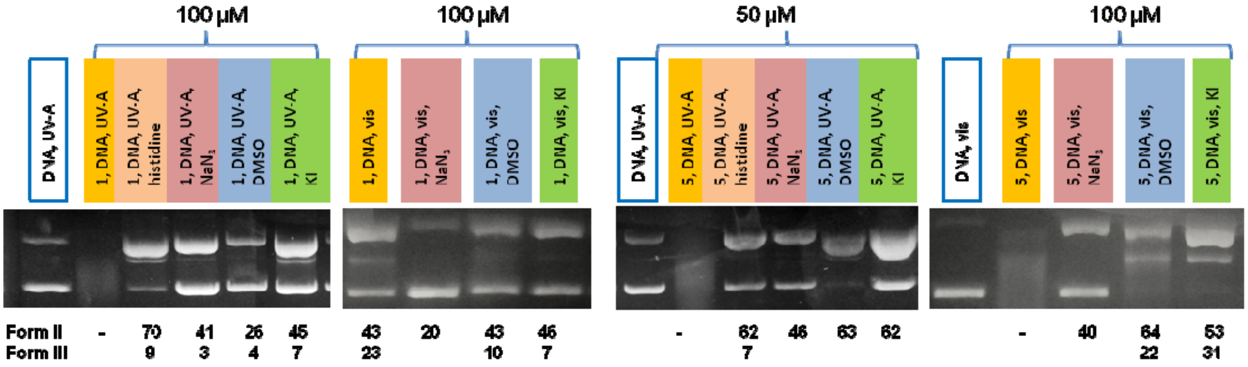

histidine: 250 µM; NaN_3: 20 mM; DMSO: 20%; KI: 250 µM

S.9 In silico Molecular Dockings for compounds 1-17 with DNA

Geometry Optimization of ligands: Gaussian 09, B3LYP/6-31g*.
DNA: 1bna (Protein Data Bank), water molecules removed, Gasteiger charges added. (ADT)
Grid Box: 60 × 80 × 114 with 0.375 Å , (whole DNA)
Docking protocol: rigid
Program: AutoDock Vina
Algorithm: Lamarkian Genetic Algorithm, runs= 100 (exhaustiveness)

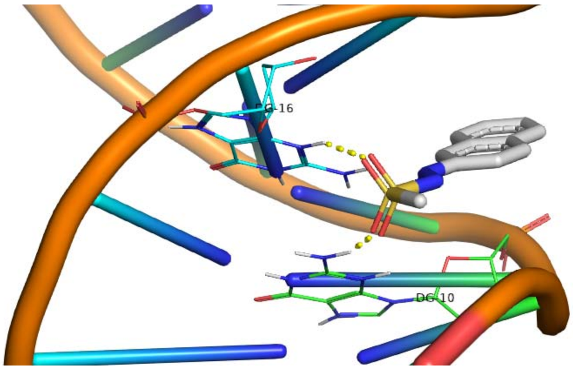

1

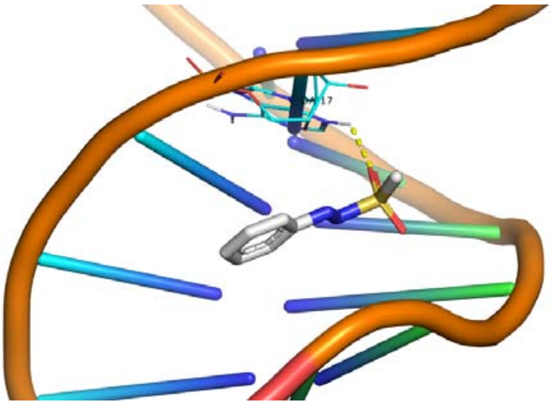

2

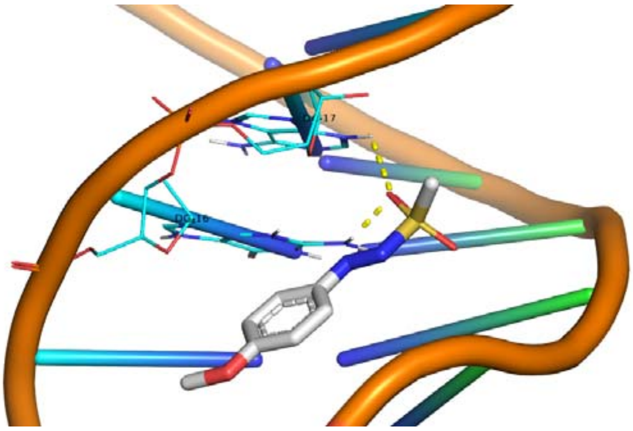

3

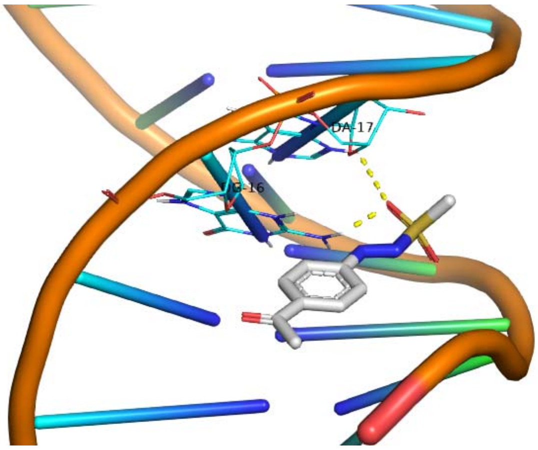

4

Figure part S.9.1 Molecular Docking calculations for the compounds of Group A.

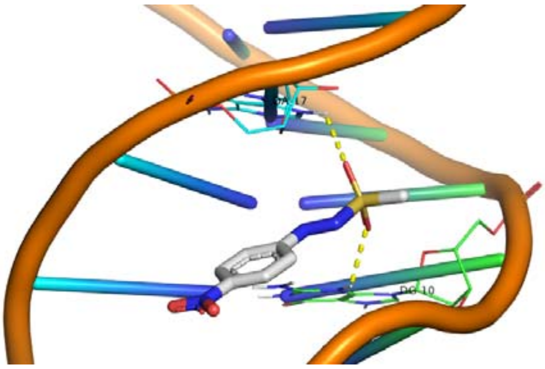

5

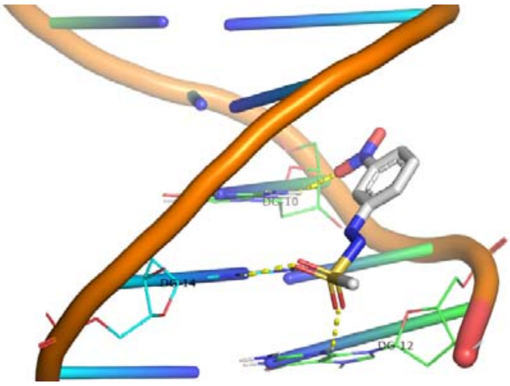

6

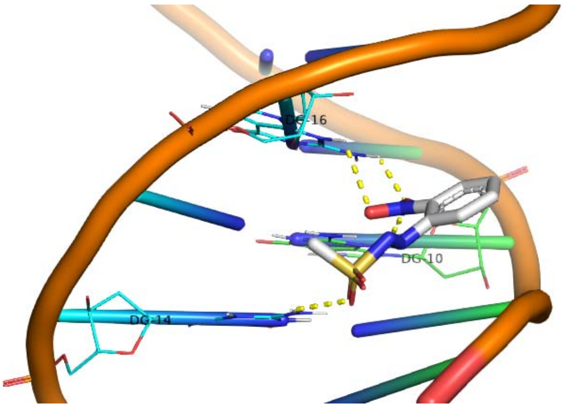

7

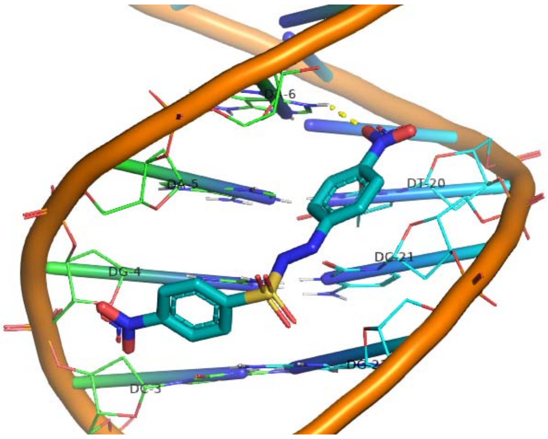

16

Figure part S.9.2 Molecular Docking calculations for the compounds of Group B.

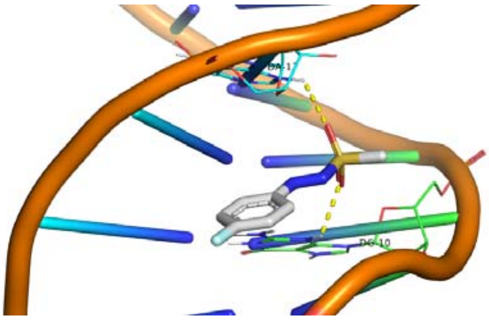

8

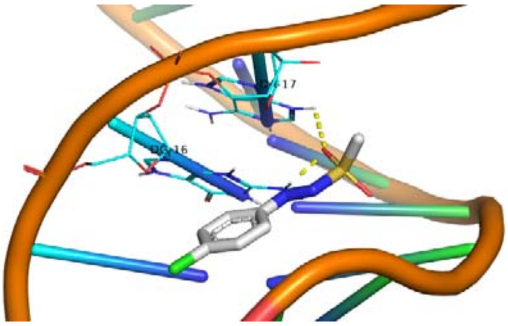

9

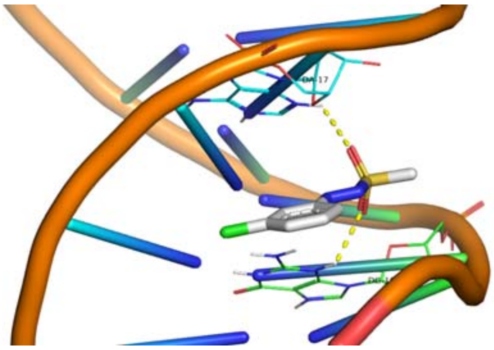

10

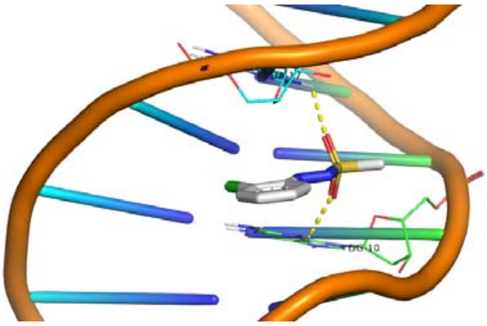

11

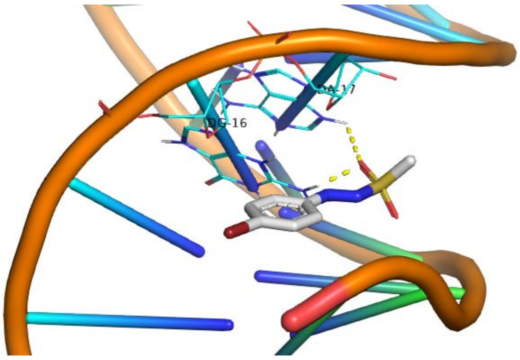

12

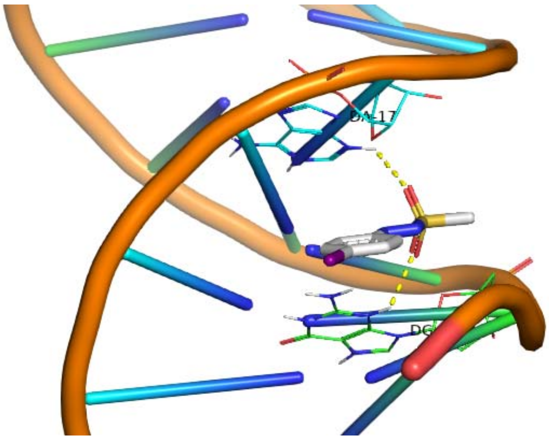

13

Figure part S.9.3 Molecular Docking calculations for the compounds of Group C.

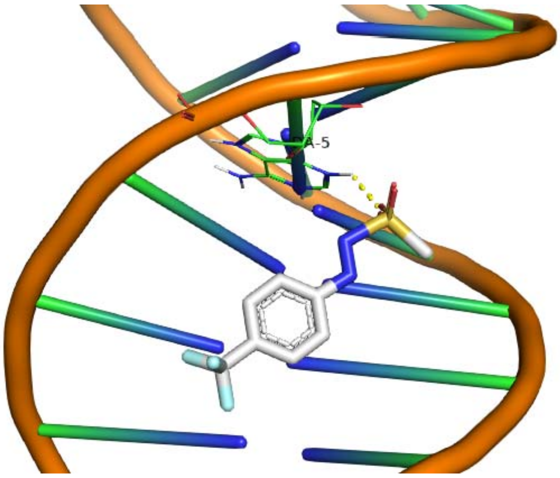

14

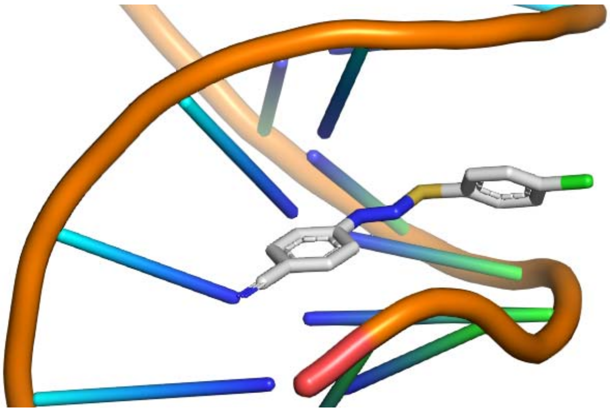

15

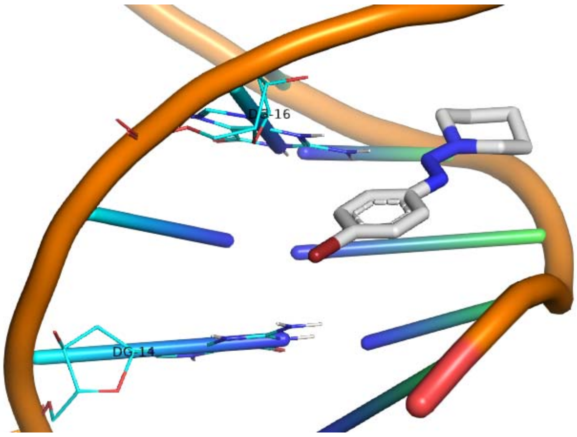

17

Figure part S.9.4 Molecular Docking calculations for the compounds of Group D.

S.10 Cell culture experiments of compounds 1-17 at concentration 50 μM

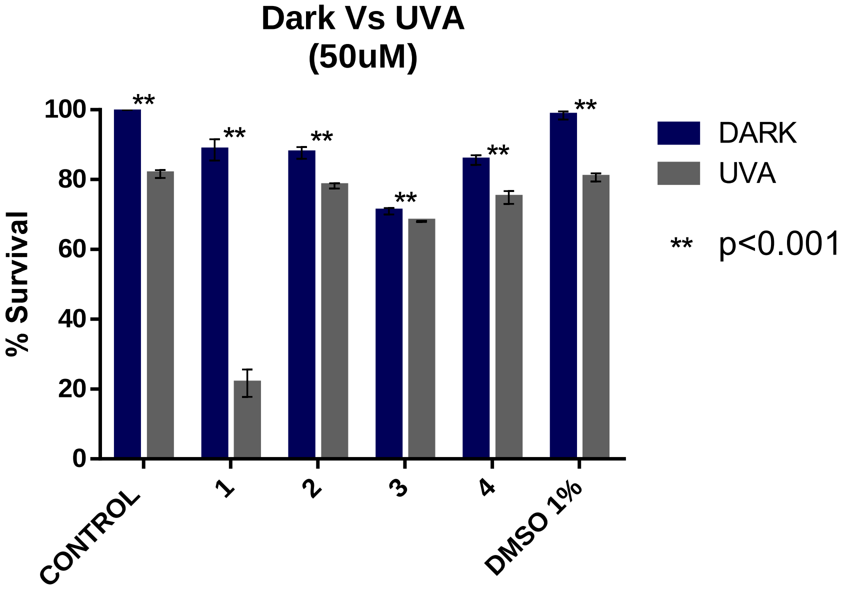

Figure part S.10.1 A375 melanoma cell culture viability experiments. Effectiveness of compounds of Group A in dark and under UV-A irradiation at 50 μM.

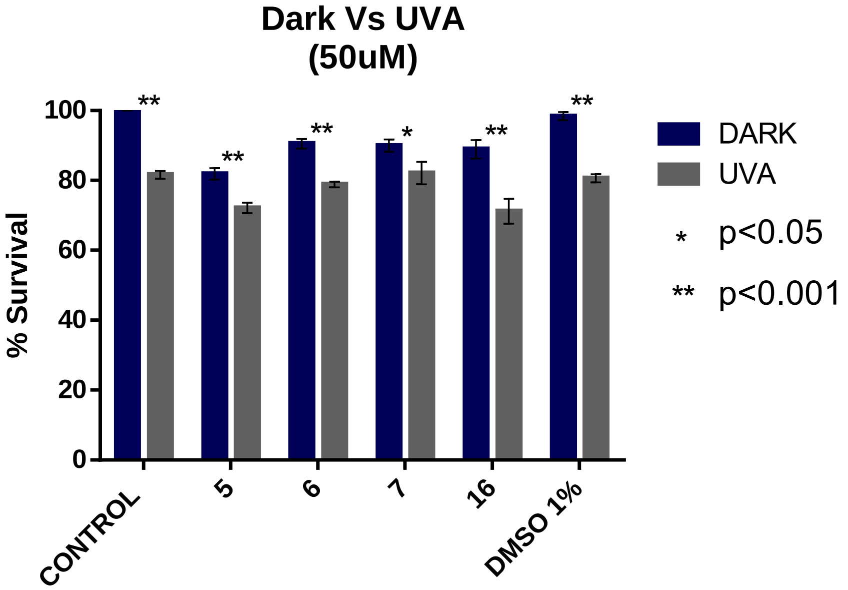

Figure part S.10.2 A375 melanoma cell culture viability experiments. Effectiveness of compounds of Group B in dark and under UV-A irradiation at 50 μM.

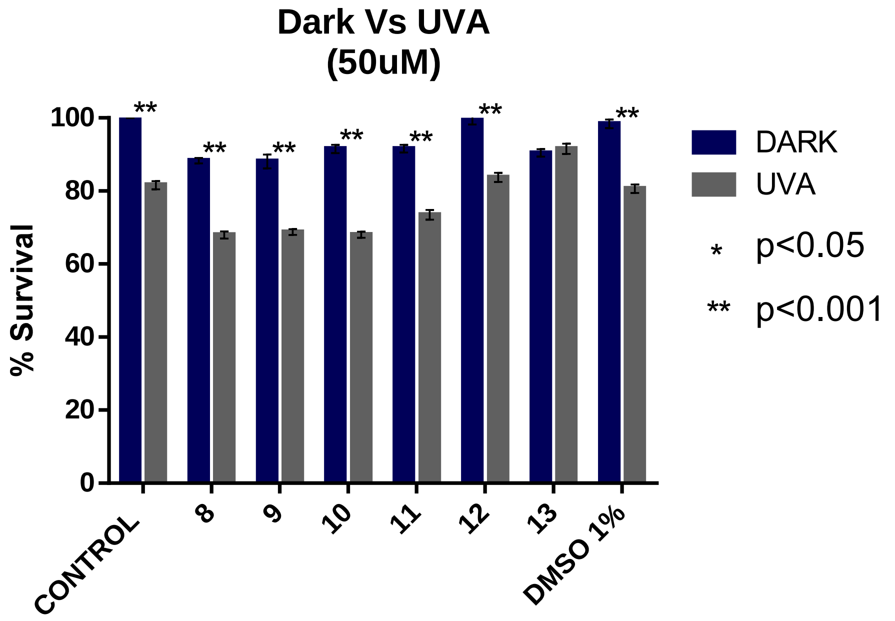

Figure part S.10.3 A375 melanoma cell culture viability experiments. Effectiveness of compounds of Group C in dark and under UV-A irradiation at 50 μM.

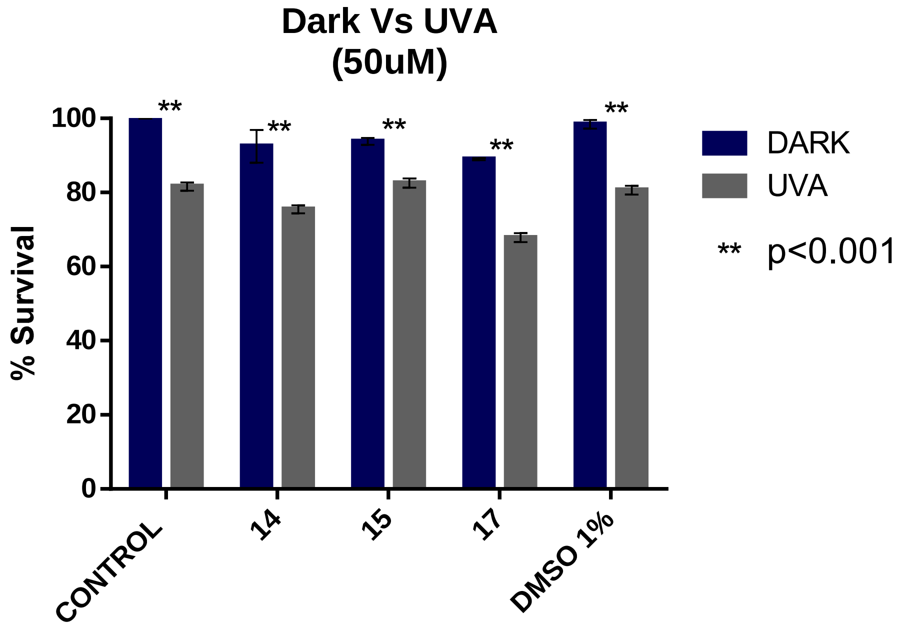

Figure part S.10.4 A375 melanoma cell culture viability experiments. Effectiveness of compounds of Group D in dark and under UV-A irradiation at 50 μM.
